# Inhibition of early EHDV2-Ibaraki infection steps in bovine cells by endosome alkalinization or ikarugamycin, but not by blockage of individual endocytic pathways

**DOI:** 10.3389/fcimb.2025.1494200

**Published:** 2025-02-06

**Authors:** Maya Malka, Inbar Czaczkes, Shlomi Kashkash, Shirel Shachar, Eran Bacharach, Marcelo Ehrlich

**Affiliations:** The Shmunis School of Biomedicine and Cancer Research, George S. Wise Faculty of Life Sciences, Tel Aviv University, Tel Aviv-Yafo, Israel

**Keywords:** EHDV2-Ibaraki, clathrin-mediated endocytosis, endosomes, ikarugamycin, entry, endocytosis

## Abstract

The Epizootic hemorrhagic disease virus (EHDV), an orbivirus, is the etiological factor of a fatal hemorrhagic disease of wild ruminants. A subset of EHDV serotypes, including the Ibaraki strain of EHDV2 (EHDV2-Ibaraki), infect and cause disease in cattle, thus posing a potential threat to livestock. As a member of the Sedoreoviridae family, the EHDV particle is devoid of a membrane envelope and is predicted to employ endocytic pathways for infection. However, the degree of dependence of EHDV2-Ibaraki on specific internalization pathways while infecting bovine cells (its natural host) is unknown. The endosome alkalinizing agent ammonium chloride blocked EHDV2-Ibaraki infection of Madin-Darby Bovine Kidney (MDBK) cells with dependence on its time of addition, suggesting the criticality of endosomal pH for the completion of early stages of infection. Treatment of cells within the alkalinization-sensitive window (i.e., before endosomal processing) with inhibitors of actin polymerization, macropinocytosis (amiloride), or dynamin GTPase activity (dynasore or dynole), or with the cholesterol-depleting agent methyl-β-cyclodextrin, failed to reduce EHDV2-Ibaraki infection. In contrast, in this same treatment time frame, ikarugamycin potently inhibited infection. Moreover, ikarugamycin inhibited interferon induction in infected cells and induced the accumulation of enlarged Rab7- and lamtor4-decorated vacuoles, suggesting its ability to block viral processing and modify late-endosome compartments. Notably, ikarugamycin treatment at initial infection stages, augmented the infection of MDBK cells with the vesicular stomatitis virus while inhibiting infection with bluetongue virus serotype 8. Together, our results point to differential antiviral effects of ikarugamycin on viruses dependent on distinct sets of endosomes for entry/processing.

## 1 Introduction

The epizootic hemorrhagic disease virus (EHDV) is an arbovirus of the genus orbivirus of the *Sedoreoviridae* family (Matthijnssens et al., 2022). EHDV is transmitted by Culicoides biting midges and is the etiological agent of the epizootic hemorrhagic disease (EHD), an acute, infectious, and possibly fatal viral disease of wild ungulates such as the North American white-tailed deer (Maclachlan et al., 2015). There are seven serotypes of EHDV, a subset of which also infect and cause disease in cattle, posing thus a potential threat to livestock with economic implications (Golender et al., 2017). Among this subset, the Ibaraki virus, subsequently classified as EHDV2 (denominated throughout this study as EHDV2-Ibaraki), was isolated from infected cattle in 1959 in Ibaraki, Japan (Inaba, 1975; Omori et al., 1969), where it caused anorexia, deglutition disorder, and miscarriages. As with other orbiviruses (e.g., the bluetongue virus, BTV), the non-enveloped EHDV virion is icosahedral and composed of three concentric protein layers, which enclose a genome composed of 10 double-stranded RNA (dsRNA) segments. These segments encode for seven structural proteins (VP1 to VP7) and the nonstructural (NS) proteins NS1 to NS4. The similitude of the structure of viruses of the *Sedoreoviridae* family correlates with similarities in interactions of these viruses with target cells (e.g., replication through transcriptionally active particles in the cytoplasm and formation of viroplasms (Papa et al., 2021; Barhoom et al., 2011; Kar et al., 2007). However, significant differences in virus-cell interactions can be observed among different strains of the same virus type. Examples of this phenomenon include the differential dependence on acidic pH and employment of distinct endocytic pathways by distinct Rotavirus strains (Gutiérrez et al., 2010; Díaz-Salinas et al., 2014) and the different entry pathways proposed for BTV in different cell types (Stevens et al., 2019; Forzan et al., 2007; Gold et al., 2010).

Endocytic pathways are classified according to different parameters, including the coating of pits and vesicles by a specific protein component [e.g., clathrin-mediated endocytosis, CME (Kaksonen and Roux, 2018; Ehrlich et al., 2004)], the degree of dependence on specific membrane components [e.g., cholesterol-dependent endocytosis (Torgersen et al., 2001)], the involvement of particular cellular factors which aid in membrane bending and/or separation of the incoming vesicle from the membrane [e.g., dynamin- (Antonny et al., 2016; Macia et al., 2006), actin- (Yarar et al., 2005; Boucrot et al., 2006; Cureton et al., 2009) or endophilin-mediated endocytosis (Boucrot et al., 2015)], or the amount of liquid that is engulfed in the process of vesicle formation [e.g., macropinocytosis (Marques et al., 2017; Swanson, 2023)]. To access the cytoplasm, viruses in general and non-enveloped viruses in particular use different endocytic pathways for the initial internalization step. This results in the localization of the incoming virus to distinct endosomal compartments, where the ignition of the latter steps of viral entry is regulated by mechanisms that include proteolytic processing [e.g., the involvement of cathepsins in the entry of the mammalian reovirus (Ebert et al., 2002)] and/or pH-dependent conformational alterations of viral entry factors [e.g., for BTV (Wu et al., 2019; Zhang et al., 2016)]. Of note, viruses differ in the endosomal compartment from which they enter the cytoplasm and are classified as early-penetrating [e.g., the vesicular stomatitis virus, VSV (White et al., 1981)] or late-penetrating viruses [e.g., BTV (Patel et al., 2016)]. While an initial assessment identified caveolin-mediated endocytosis as a viral entry pathway that bypasses endosomal compartments, this has been reassessed, and the majority of endocytic mechanisms are thought to deliver incoming viruses to components of the endo-lysosome system of the cell (Engel et al., 2011; Donaldson, 2019). Of note, the endo-lysosomal compartments of the cell also serve as platforms for recognizing incoming viruses and activating antiviral responses. For example, the Toll-Like Receptor 3 (TLR3), which senses and responds to dsRNA, localizes to and initiates signaling in endosomes in a pH-dependent manner (de Bouteiller et al., 2005) and was proposed to depend on CME (Itoh et al., 2008).

Endocytosis in general, and viral entry in particular, have been studied via pathway-selective inhibition [e.g (Abu Rass et al., 2022)]. Methods of inhibition include cholesterol depletion (Ehrlich et al., 2004; Grimmer et al., 2002; Torgersen et al., 2001), inhibition of actin dynamics (Boucrot et al., 2006; Yarar et al., 2005; Boulant et al., 2011) or of the GTPase activity of dynamin (Macia et al., 2006; Antonny et al., 2016), or inhibitors with uncharacterized molecular targets such as the inhibition of CME by ikarugamycin [IKA (Elkin et al., 2016)]. Compounds that alter the physicochemical parameters of the cell, such as an alteration to cytosolic pH with amiloride, may inhibit specific pathways, e.g., micropinocytosis (Koivusalo et al., 2010). Notably, and in line with proposed functions for the ensemble of endocytic pathways in maintaining membrane homeostasis, inhibition of a given pathway may result in the upregulation of another (Damke et al., 1995). Given that the entry pathway used by EHDV2-Ibaraki to infect bovine cells remains undescribed, we employed different inhibitory treatments to dissect the cellular requirements for EHDV2-Ibaraki entry and sensing in Madin-Darby Bovine Kidney (MDBK) cells.

## 2 Materials and methods

### 2.1 Cell lines

Madin-Darby bovine kidney (MDBK) cells (a kind gift from the Kimron Veterinary Institute, Beit-Dagan, Israel). Cells were checked for absence of Bovine Viral Diarrhea Virus (BVDV) and were cultured in Eagle’s Minimum Essential Medium (EMEM) supplemented with 10% of non-inactivated horse serum (HS), 2 mM L-Glutamine, 100 U/ml penicillin, 100 µg/ml streptomycin, and nonessential amino acids (Cat. number: 01-340-1B) (All from Biological Industries, Israel). Spontaneously immortalized ovine kidney (OK) cells and Vero African Green Monkey Kidney cells were cultured and employed in viral passaging and plaque assays as previously described in (Shai et al., 2013; Dellac et al., 2021).

### 2.2 Inhibitors

The inhibitors’ sources and final concentrations are indicated in brackets: ammonium chloride (Sigma-Aldrich, A9434; 25 mM), dynasore (Sigma-Aldrich, D7693; 80 μM), dynole 34-2 (Abcam, ab120463; 40 μM), ikarugamycin (Cayman Chemical, 15386; 0.5 μM, 2 μM), methyl-β-cyclodextrin (Sigma-Aldrich, 332615; 15 mM), amiloride (Sigma-Aldrich, 1019701; 1 mM), latrunculin-B (Abcam, ab144291; 1 μM).

### 2.3 Fluorescent probes

4’,6-diamidino-2-phenylindole (DAPI, Invitrogen, D1306), Alexa Fluor 568 Phalloidin (Invitrogen, A12380), Lysotracker deep red (Invitrogen, L12492).

### 2.4 Viruses

Epizootic hemorrhagic disease virus serotype 2-EHDV2-Ibaraki was obtained and passaged as in (Shai et al., 2013). We have previously described the generation of the VSV mutant VSV-ΔM51 (Dellac et al., 2021). The same procedure was used to recover the GFP-expressing VSV clone (VSV-GFP) (Andreu-Moreno and Sanjuán, 2018), generously provided by Dr. Ron Geller (Institute for Integrative Systems Biology (I2SysBio), Universitat de Valencia-CSIC, Valencia, Spain). Bluetongue virus serotype 8 (BTV-8) was a kind gift from Prof. Eyal Klement (Koret School of Veterinary Medicine, Hebrew University of Jerusalem, Israel).

### 2.5 UV inactivation of virus

EHDV2-Ibaraki ( $1.2\times 10^{7}$ PFU/ml) was irradiated as described in (Shai et al., 2013).

### 2.6 Quantification of viral titers

For EHDV2-Ibaraki and BTV-8, viral titers were quantified by plaque assays previously described (Shai et al., 2013). For VSV infections, plaque assay was performed as previously described (Dellac et al., 2021).

### 2.7 Protein extraction and immunoblotting

The cell pellet was resuspended in cold RIPA solution (10 mM Tris-HCl pH 7.5, 1 mM EDTA, 0.5 mM EGTA, 1% Triton X-100, 0.1% Deoxycholate, 0.1% SDS, 140 µM NaCl) supplemented with 1:25 protease inhibitor cocktail (Sigma-Aldrich) and 1:100 phosphatase inhibitor was cocktails I and II (Sigma-Aldrich) and incubated at 4°C for 1 hour. Lysed cells were centrifuged at 13,000 rpm, 4°C for 13 minutes. Supernatants were collected, and protein concentration was determined via Bicinchoninic acid (BCA) assay (Cyanogen). Equal amounts of protein extracts (30 µg) in sample buffer containing β-mercaptoethanol were separated via sodium dodecyl sulfate-polyacrylamide gel electrophoresis (SDS-PAGE) through a 10% polyacrylamide gel and subsequently transferred onto a nitrocellulose membrane (Bio-Rad). Membranes were blocked in 5% skim milk dissolved in TTBS (20 mM Tris-HCl pH 7.5, 15 mM NaCl, 0.1% TWEEN20) for 1 hour at room temperature and then incubated with a primary antibody for 1 hour at room temperature. Next, membranes were incubated with a secondary antibody linked to HRP in 5% skimmed milk for 1 hour at room temperature. Immunoreactive bands were detected by chemiluminescence. Densitometry of the immunoblots was quantified using the Image Lab program.

### 2.8 Antibodies

Monoclonal mouse anti-nonstructural protein 3 of EHDV2-Ibaraki antibody (anti-NS3) was previously described (Shai et al., 2013)). The α-Myc tag hybridoma (9E10) was a generous gift from Prof. Yoav Henis (Tel Aviv University). Commercial antibodies included monoclonal mouse anti-HSP-70 (Sigma-Aldrich, H5147), monoclonal mouse anti-β-Actin (Merck, A2228), and monoclonal rabbit anti-LAMTOR4 (Cell Signaling, 13140). Secondary antibodies included HRP-conjugated goat anti-mouse IgG (Jackson ImmunoResearch, 115-035-146), goat-anti-mouse and goat-anti-rabbit antibodies labeled with Alexa-488 or Alexa-647 respectively (Invitrogen).

### 2.9 qRT-PCR

Total RNA was extracted from cells using the EZ-RNA kit (Biological Industries Beit Haemek, 20-400-100), followed by reverse transcription using the q-PCR-BIO cDNA Synthesis Kit (q-PCR-BIO, PB30.11-10) according to the manufacturer’s instructions, with additional no-RNA control. Real-Time PCR analysis of the mRNA levels of IFNβ, NS3, and GAPDH was done in triplicates, using Fast SYBR-green master mix (Bio-Rad CFX connect) with BIO-RAD Real-Time PCR System (Applied Biosystems, 4376600). The following RT-qPCR primers were used: bovine IFNβfw 5’AAGACTCAGCTTCAGCACCTAC3’, bovine IFNβrev 5’ACACTCTTTAAGGCTCTGACG3’, NS3fw 5’CACGCCAACATCAATGCCAA3’, NS3rev 5’TGACGCATACGCAACCTTCT3’, BTV8fw 5’GCATAAAATACTAGAGGATGGCG3’, BTV8rev 5’AGCYGTTCCAATCACAACC3’ bovine GAPDHfw 5’AAGGTCGGAGTGAACGGATTC3’, bovine GAPDHrev 5’ATGGCGACGATGTCCACTTT3’.

### 2.10 Microscopy

Imaging was performed with two systems: 1- Axiovert 200M (Carl Zeiss MicroImaging) controlled by SlideBook 5.0 (Intelligent Imaging Innovations), employing either a 63x oil immersion objective (Plan Apochromat, NA 1.4) or a 10x air objective (Plan Apochromat, NA 0.25) and registered on an EZ camera (Photometrics). 2- EVOS M5000 Imaging System (Invitrogen) employing either 10x air or 60x oil immersion objectives.

### 2.11 Fluorescence microscopy experiments

Transferrin internalization. 2x10^5^ MDBK cells were plated on 13 mm-glass coverslips. Cells were pre-treated (or not) with the different inhibitors (30 min, in serum-free medium). Incubation with transferrin Alexa Fluor 488 Conjugate (Invitrogen, T13342, 50 μg/ml) was for 20 min at 37°C, in the same medium as the pre-treatment. Fixation was with 4% paraformaldehyde (PFA), after which cells were permeabilized in PBT solution (2% BSA, 0.1% Triton X-100 in PBS, 5 min RT) and stained with DAPI (45 minutes, in PBT). Mounting was with the fluorescent mounting medium (Getter; E18-18).

Endocytosis of Myc-labeled type II transforming growth factor-β receptor (Myc-TβRII). COS7 cells were grown on glass coverslips and transfected with Myc-TβRII in pcDNA3. 24 h after transfection cells were labeled in the cold with anti-Myc IgG [as in (Chetrit et al., 2011)]. Cells were either kept on ice or warmed to 37°C for different time periods, after which they were fixed and permeabilized as described above. Subsequently, cells were permeabilized and stained with secondary antibodies and DAPI prior to mounting.

Staining of LAMTOR4. 2x10^5^ MDBK cells grown on glass coverslips were treated or not with IKA (1 µM, 20 h) prior to fixation, permeabilization, and staining as described above.

For staining of acidic organelles, 2x10^5^ MDBK cells were plated in a 12-well plate, treated or not with IKA (2 μM, 24 h), and incubated with lysotracker deep red (Invitrogen, L12492, 30 μM, 25 min, 37°C).

Acid wash, for the removal of membrane-localized transferrin or α-myc antibodies, was performed by repeated cold washes with acidic glycine buffer (150 mM NaCl, 0.1M Glycine, pH 2.5) prior to fixation.

### 2.12 Transfection and plasmids

Transfections were performed with lipofectamine 2000 (Invitrogen, 11668019) in Opti-MEM (Gibco, 31985062) according to the manufacturer’s protocol. The plasmids encoding for LCA-GFP, Rab5-GFP, and Rab7-GFP were kind gifts by Tomas Kirchhausen (Harvard University). Myc-tagged TβRII has been previously described (Ehrlich et al., 2001). For GFP-fusion markers, after 12h of transfection, the cells were treated or not with IKA (1 μM) for 24 h. For endocytosis experiments with myc-TβRII, at 16 h-post transfection, cells were pre-treated (or not) with IKA (2 µM, 30 min) before labeling and internalization steps.

### 2.13 Image analysis

To quantify the fluorescence intensity of cell-associated fluorescent transferrin, cell fields were imaged in three dimensions (24 stacks, 0.16 μm spacing between stacks). Image stacks were deconvolved (NearestNeighbours option, SlideBook 5.0) and projected on a 2D image. Signals were segmented according to intensity thresholds for the DAPI and FITC channels. The sum intensity of FITC was normalized to the cell number by dividing it with the sum intensity of the DAPI signal.

To quantify the percentage of VSV-infected cells, fields of infected cells were imaged with a 10x objective. The number of cells in the field and the VSV-positive cells were assessed via intensity-based segmentation and size-based object definition, employing Slidebook™. Quantification of the percentage of cells exhibiting LAMTOR vacuoles was performed via manual inspection of multiple fields acquired with a 60x objective.

### 2.14 Statistical analysis

One-way ANOVA and Dunnett’s test for multiple comparisons were applied in experiments comprising a single control and multiple experimental conditions. For comparisons of one treatment and its control, a two-tailed t-test was performed, assuming equal variance between groups. A P value of ≤ 0.05 was considered significant. All statistical analysis was done using the GraphPad Prism software.

## 3 Results

### 3.1 Endosome alkalinization with NH_4_Cl blocks EHDV2-Ibaraki infection in MDBK cells

**Figure 1:**
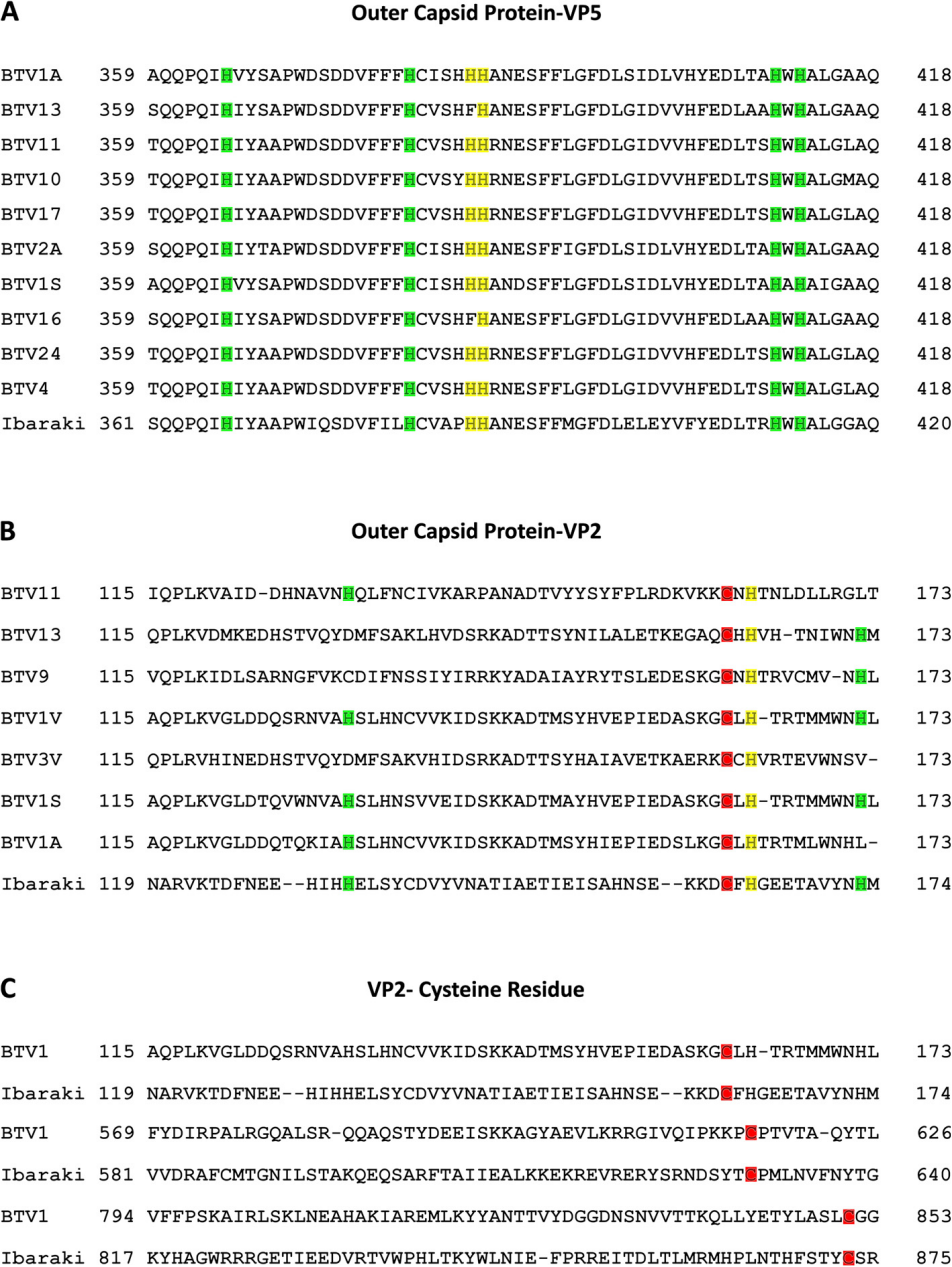
Conservation of functional residues in VP5 and VP2 proteins of EHDV2-Ibaraki and in BTV counterparts. Protein sequence alignment of selected segments of the VP5 and VP2 proteins of EHDV2-Ibaraki with the analogous proteins from different BTV serotypes. Sequences were retrieved from UniProt and aligned with NCBI protein BLAST (Sayers et al., 2022). **(A)** Alignment of a segment of the VP5 protein of EHDV2-Ibaraki with analogous segments of VP5 from 10 different BTV serotypes. Color-coding marks conserved histidine residues in EHDV2-Ibaraki and the different BTV serotypes. Marked in yellow are H_385_ and H_386_, which are proposed as pH sensors in BTV-VP5 (Wu et al., 2019; Hassan and Roy, 1999). Marked in green are additional histidine residues exhibiting conservation. **(B)** Alignment of a segment of the VP2 protein of EHDV2-Ibaraki with the analogous protein of 7 BTV strains. Color coding is as in **(A)** the proposed pH sensor H_164_ is in yellow, and additional conserved histidines are marked in green. Also marked is C_162_ (red), corresponding to one of the three cysteines proposed to form a pH-sensing zinc finger (Wu et al., 2019). **(C)** Alignment of cysteines that form the pH-sensing zinc finger in BTV1 and are conserved in EHDV2-Ibaraki. C_162_, C_630_, and C_873_ (EHDV2-Ibaraki) and C_162_, C_617_, and C_851_ (BTV1), are marked in red.

Within the orbivirus genus of the Sedoreoviridae family, Bluetongue, and Epizootic Hemorrhagic Disease viruses exhibit a considerable sequence similarity. Comparison of the sequences of EHDV2-Ibaraki with those of 15 BTV serotypes (summarized in **Table 1**) showed average identity values at the amino acid level for 10 structural and non-structural proteins (VP1-7, NS1-3) ranging from 23% (for VP2) to 80% (for VP3), and average residue similarity values ranging from 42% (for VP2) to 91% (for VP3). The entry of BTV into mammalian cells involves the VP2 and VP5 proteins (Forzan et al., 2007; Zhang et al., 2010), with critical pH-sensing functions being performed by conserved histidines (H_385_-H_386_ in BTV-VP5 and H_164_ of BTV-VP2) and cysteines in BTV-VP2 (C_162_, C_617_, C_851_). The latter, together with H_164_, form a Zinc finger (Wu et al., 2019). These conserved residues mediate conformation alterations to VP2 and VP5 following protonation in acidic endosomal compartments (Hassan and Roy, 1999; Wu et al., 2019). Comparison of the VP2 and VP5 proteins of EHDV2-Ibaraki with those of 10 BTV serotypes (for VP5) or 7 BTV serotypes (for VP2) revealed alignment of H_387_ and H_388_ of EHDV2-Ibaraki-VP5, and H_164_ of EHDV2-Ibaraki-VP2, align with cognate histidine residues in BTV-VP5 and BTV-VP2 (**Figure 1**). Moreover, the three Zinc-finger-associated cysteines of VP2 (C_162_, C_617_ and C_851_ in BTV1), are also conserved in EHDV2-Ibaraki (C_162_, C_630_ and C_873_). Based on this, we hypothesized that EHDV2-Ibaraki requires endosome acidification for infection of MDBK cells and tested this by treating cells with NH_4_Cl (25 mM) 30 minutes before and throughout infection (MOI = 1, 24 h). To visualize the levels of infection and the portion of infected cells, we labeled uninfected, infected, and infected/NH_4_Cl-treated cells against the nonstructural protein 3 (NS3) of EHDV2-Ibaraki, and imaged them by fluorescence microscopy. This revealed a complete lack of NS3 expression in cells treated with NH_4_Cl, indicative of a complete inhibition of infection (**Supplementary Figure 1**).

**Table 1:** Percentage of identities and similarities of EHDV2-Ibaraki with different BTV strains.

		VP1	VP2	VP3	VP4	VP5	VP6	VP7	NS1	NS2	NS3
**BTV1**	Identities	73%	23%	80%	66%	60%	47%	64%	50%	49%	55%
	Positives	85%	44%	91%	80%	77%	59%	81%	68%	63%	73%
**BTV2**	Identities	74%	23%	79%	65%	59%	45%	64%	51%	48%	55%
	Positives	85%	42%	91%	79%	76%	61%	82%	69%	62%	73%
**BTV3**	Identities	73%	22%	80%	66%	58%	48%	65%	50%	51%	54%
	Positives	85%	42%	91%	80%	76%	63%	81%	69%	64%	73%
**BTV4**	Identities	73%	24%	80%	66%	59%	48%	64%	51%	51%	54%
	Positives	85%	43%	91%	80%	76%	64%	82%	68%	64%	71%
**BTV5**	Identities	73%	25%	80%	66%	59%	47%	64%	51%	51%	55%
	Positives	85%	42%	92%	80%	76%	64%	82%	68%	64%	73%
**BTV7**	Identities	73%	23%	80%	66%	58%	49%	62%	50%	51%	55%
	Positives	85%	42%	91%	80%	75%	63%	80%	68%	64%	73%
**BTV9**	Identities	73%	22%	80%	66%	58%	48%	64%	51%	50%	56%
	Positives	85%	42%	91%	80%	75%	62%	82%	68%	63%	74%
**BTV10**	Identities	73%	23%	80%	66%	59%	48%	64%	51%	51%	54%
	Positives	84%	42%	91%	80%	75%	63%	82%	68%	64%	72%
**BTV11**	Identities	73%	23%	80%	66%	59%	48%	64%	51%	48%	52%
	Positives	85%	42%	91%	80%	76%	63%	82%	68%	62%	68%
**BTV12**	Identities	73%	23%	80%	66%	60%	48%	64%	51%	49%	54%
	Positives	85%	42%	91%	80%	78%	63%	82%	68%	62%	72%
**BTV13**	Identities	73%	24%	80%	65%	59%	48%	64%	51%	51%	55%
	Positives	85%	43%	91%	79%	77%	63%	81%	68%	64%	73%
**BTV16**	Identities	74%	23%	80%	66%	59%	49%	64%	51%	50%	56%
	Positives	85%	41%	91%	80%	76%	63%	81%	68%	63%	73%
**BTV18**	Identities	73%	21%	80%	66%	59%	48%	64%	51%	51%	54%
	Positives	85%	40%	91%	80%	78%	63%	81%	68%	64%	72%
**BTV21**	Identities	73%	24%	80%	66%	59%	49%	64%	51%	49%	55%
	Positives	85%	43%	91%	80%	76%	65%	81%	68%	63%	74%
**BTV23**	Identities	73%	22%	80%	66%	58%	48%	64%	51%	50%	56%
	Positives	85%	40%	91%	80%	76%	61%	82%	68%	63%	74%

The table summarizes the percentages of residue identity between the protein sequences (VP1-7 and NS1-3). Sequences were retrieved with UniProt and aligned with Protein Blast of NCBI. The bold values are the virus serotypes.

### 3.2 Time-dependent inhibition of infection by NH_4_Cl

**Figure 2:**
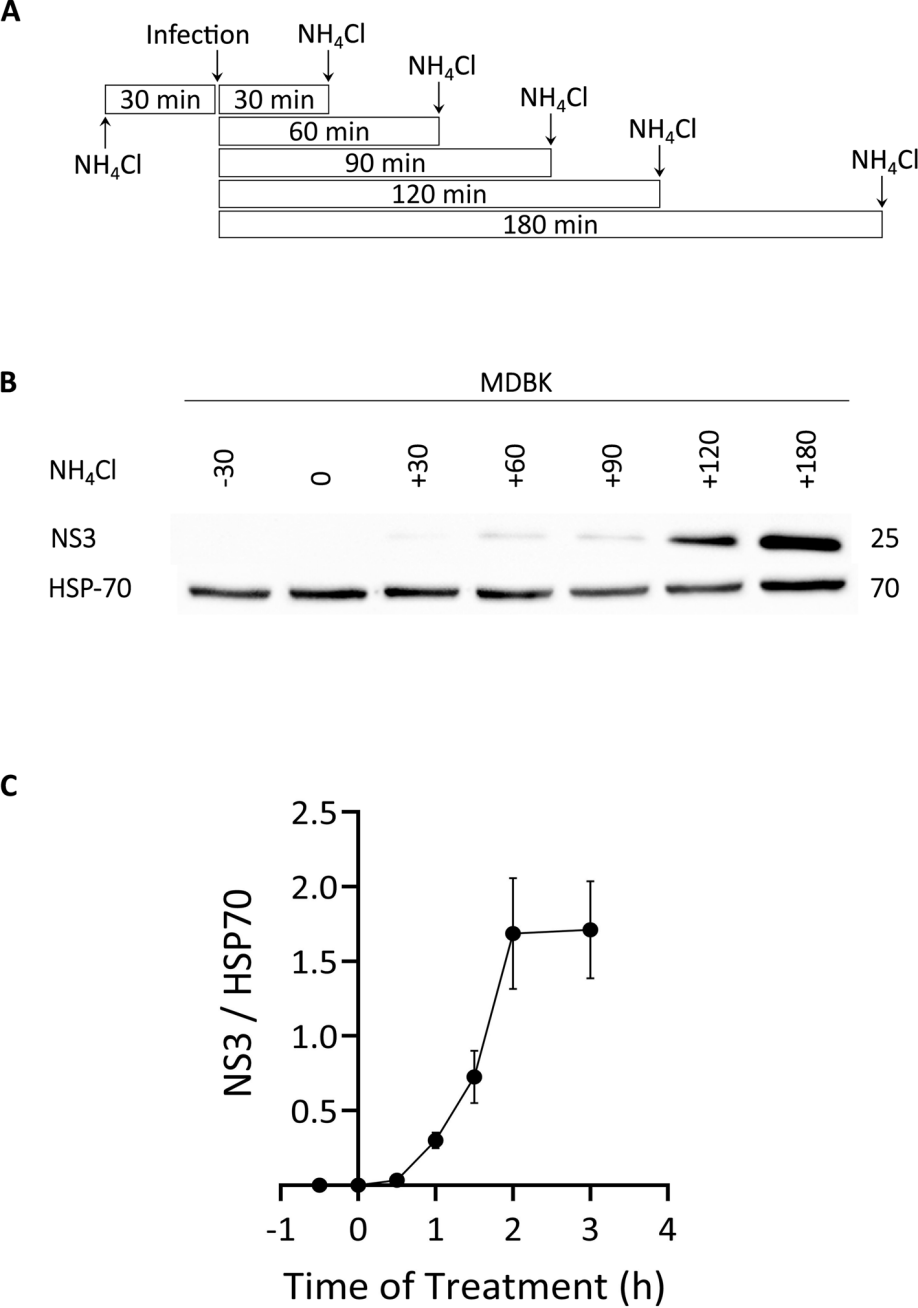
Time-dependence of NH_4_Cl-mediated inhibition of EHDV2-Ibaraki infection. NH_4_Cl (25 mM) was added at 30-minute intervals, beginning with 30 min before infection. Once added, NH_4_Cl was present for the remainder of the infection (24 h). MDBK cells were infected with EHDV2-Ibaraki (MOI = 1) at time “0”. **(A)** Schematic depiction of the timeline of NH_4_Cl addition and infection. **(B)** A representative immunoblot of the experiment described in **(A)** NS3 is a non-structural viral protein and serves as an indicator of the infection levels; heat shock protein 70 (HSP-70) serves as loading control. **(C)** The graph depicts the average ± SEM of the ratio of NS3 to HSP-70 signals at the different time points (n=5).

Next, we assessed the dependence of the inhibition of EHDV2-Ibaraki infection on the time of NH_4_Cl addition. For this, MDBK cells were treated with NH_4_Cl (25 mM) at 30-minute intervals relative to challenge with EHDV2-Ibaraki (MOI=1). Times of addition NH_4_Cl ranged from 30 min pre-treatment and included 0, 30, 60, 90, 120, or 180 min relative to the time of addition of virus (see schematic depiction, **Figure 2A**). Infection levels were assessed by examining NS3 expression at 24 hours post-infection (hpi), a time frame required for reliable detection under these experimental conditions. **Figure 2B** shows a representative immunoblot of this experiment, while the graph in **Figure 2C** summarizes multiple repeats. NS3 expression was undetectable when NH_4_Cl was added 30 min before, together with, or 30 min following EHDV2-Ibaraki challenge. The addition of NH_4_Cl at later time points (up to 2 h) resulted in progressive increase in infection in multiple experiments, while a lesser increase was observed between the 2 or 3-hour time point of NH_4_Cl addition. These results suggest that the critical step inhibited by NH_4_Cl occurs within the initial 2-3 hours of infection. We interpret these data as indicating that this is the time frame required for the virus to undergo endocytosis and pH-dependent endosomal processing.

### 3.3 Ikarugamycin, but not inhibition of individual endocytic pathways, inhibits early stages of EHDV2-Ibaraki infection

**Figure 3:**
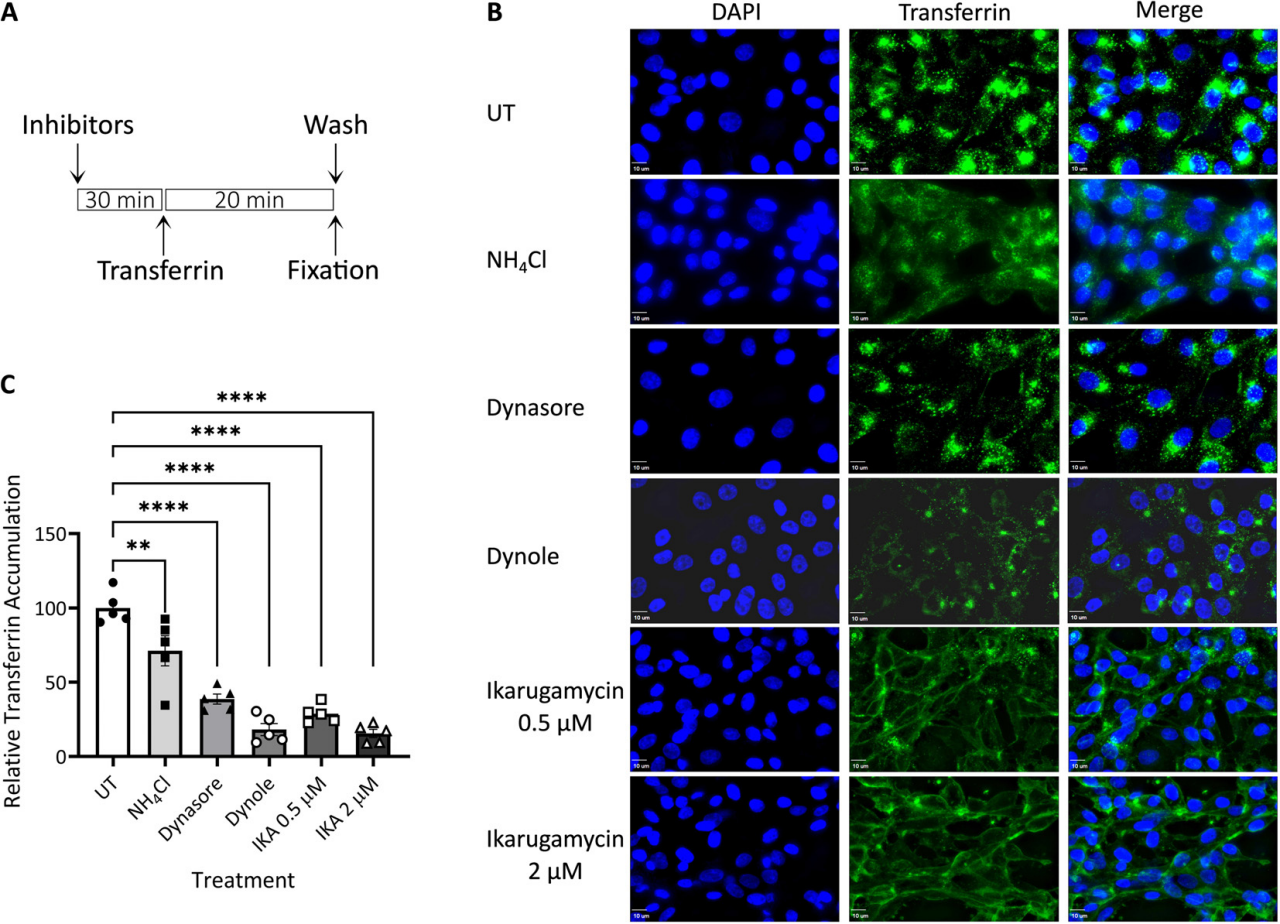
Transferrin uptake. MDBK cells were pre-treated with NH_4_Cl (25 mM), dynasore (80 μM), dynole (40 μM), or Ikarugamycin (0.5 or 2 μM), all in serum-free medium for 30 min. Cells were subsequently incubated with transferrin (50 μg/ml, 20 min; green signal) in the same medium that was employed for the pre-treatment. Following fixation-permeabilization, cells were stained with DAPI (blue signal) and imaged with a fluorescence microscope as described in the Methods section. **(A)** Depiction of the experimental timeline. **(B)** Typical 2-dimensional projections of cells under the different treatments. Bars = 10μm. **(C)** The graph depicts the average ± SEM normalized accumulation of transferrin (transferrin signal/DAPI signal) relative to the average accumulation of transferrin in untreated cells (taken as 100%). Significance was calculated by One-Way ANOVA. **p = 0.0034; ****p < 0.0001). For each condition, a minimum of 5 fields (~40 cells) were employed.

Based on the above experiments, we termed the stages preceding the NH_4_Cl-sensitive step, as early/entry stages of EHDV2-Ibaraki infection. To inquire about the dependence of these steps on the functionality of specific endocytic pathways/mechanisms, we employed a selection of inhibitors that target such pathways/mechanisms. To focus on the putative effects of such inhibitors on the early/entry stages of infection and to limit potential toxicity effects elicited by such inhibitors, we performed experiments in which cells were pretreated for 30 minutes with the inhibitors alone, followed by an hour in which cells were concomitantly exposed to virus and inhibitors, after which both inhibitor and virus were washed away before incubation of the cells with NH_4_Cl (24 h), to impede any further entry while allowing for expression of viral components that serve as indicators of infection. Repeated experiments (summarized in **Supplementary Figure 2**), with different readouts of infection (e.g., expression of NS3 protein as measured by immunoblotting, or infectious virion production as measured by plaque assay) revealed a lack of effects for (i) cholesterol depletion with methyl-β-cyclodextrin (15 mM), (ii) amiloride (1 mM), (iii) latrunculin (1 μM). To confirm the action of latrunculin, we stained cells with phalloidin at 30 minutes (corresponding to the time point of virus addition) or 90 minutes (corresponding to the time of washout). This revealed marked alterations to the phalloidin staining, in accord with the proposed function of latrunculin (**Supplementary Figure 3**). These results suggest a lack of dependence on macropinocytosis (or other actin-dependent pathways) or cholesterol-dependent endocytic pathways for EHDV2-Ibaraki entry into MDBK cells. As such, we opted to focus further analysis on inhibitors of clathrin-mediated endocytosis (CME): IKA (Elkin et al., 2016), which functions via an uncharacterized mechanism, and inhibitors of the GTPase activity of dynamin, dynasore (Macia et al., 2006) and dynole (Hill et al., 2009). The rationale for choosing two distinct dynamin inhibitors stems from their different structures. Before analyzing these inhibitors’ effects on EHDV2-Ibaraki entry, we initially assessed their effects on the internalization of fluorescently labeled transferrin, employed here as a CME marker. MDBK cells were pretreated with the inhibitors for 30 min (in serum-free medium), after which the cells were incubated with naïve or inhibitor-containing serum-free medium, supplemented with Alexa-488-labeled transferrin (50 μg/ml, 20 min, 37°C) (**Figure 3**). Quantification of cell-associated transferrin signal (normalized to DAPI signal, as an indicator of the number of cells per field, **Figure 3C**) revealed marked inhibition in the accumulation of transferrin in cells treated with the dynamin inhibitor dynole or 2 μM IKA (> 80% inhibition), suggesting their ability to potently inhibit CME. Partial reductions were induced by 0.5 μM IKA (~ 70%), dynasore (~ 60%) or NH_4_Cl (~ 30%). Notably, in the presence of IKA, a transferrin signal along the contours of the cell was observed, in line with its accumulation at the plasma membrane. To focus on internalized transferrin, we probed for the effects of acid wash before fixation and imaging, which revealed that in untreated conditions, most transferrin signals corresponded to internalized transferrin. In contrast, acid wash eliminated the remaining transferrin signal in IKA-treated cells, indicative of a complete block in transferrin internalization (**Supplementary Figure 4**). To confirm the CME-inhibitory effect of IKA, we tested its ability to inhibit the endocytosis of the Myc-tagged type II TGF-β receptor [Myc-TβRII, shown by use to occur through CME (Ehrlich et al., 2001; Hirschhorn et al., 2012)]. Here, too, 2 μM IKA sufficed to block endocytosis (**Supplementary Figure 5**).

**Figure 4:**
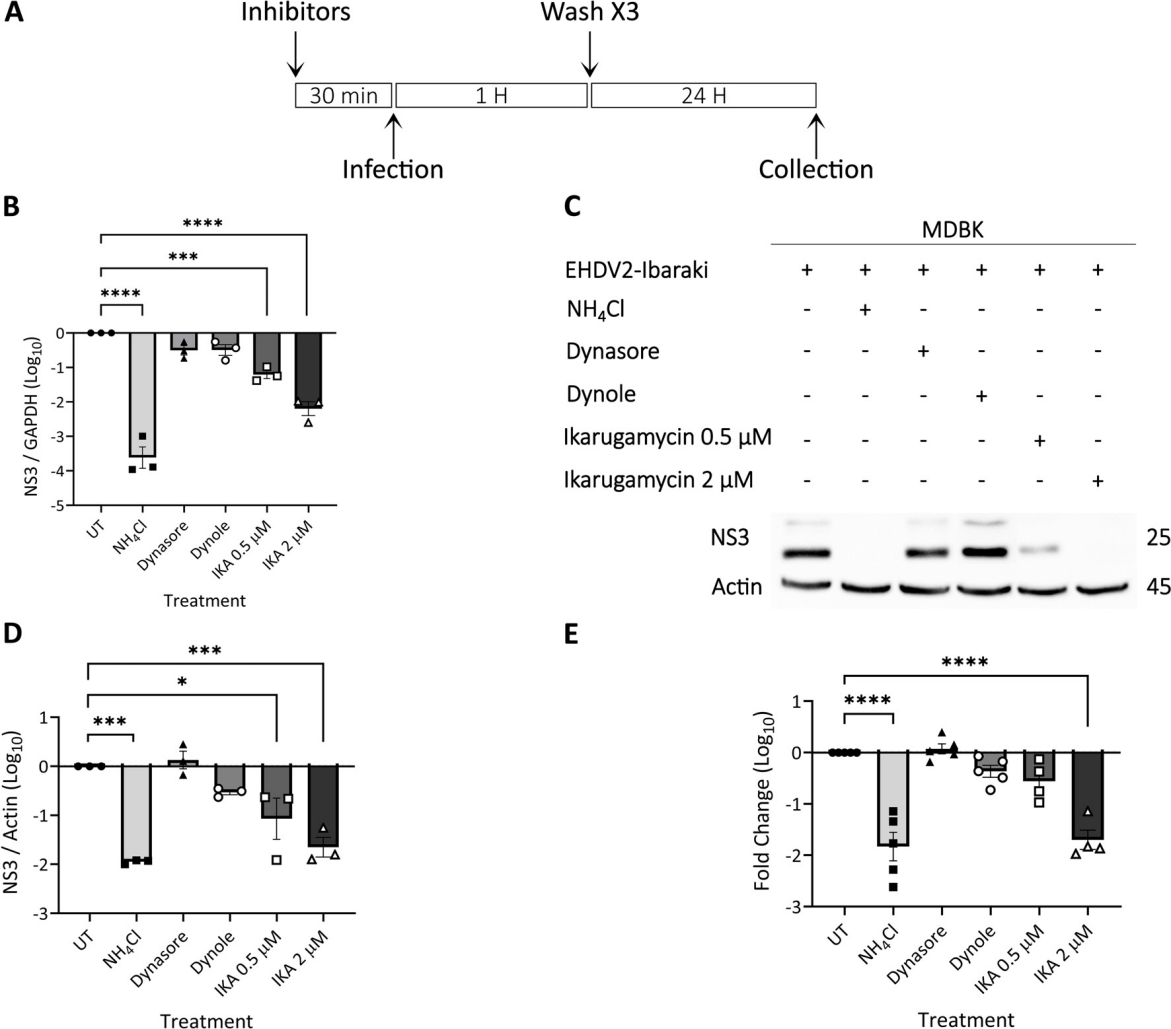
Ikarugamycin inhibits EHDV2-Ibaraki entry. MDBK cells were pre-treated with NH_4_Cl (25 mM), dynasore (80 μM), dynole (40 μM), or Ikarugamycin (abbreviated in graphs as IKA, 0.5 or 2 μM) for 30 min, followed by infection with EHDV2-Ibaraki (MOI = 1, 1 h) in the presence of the inhibitors. Following extensive washes with PBS supplemented with NH_4_Cl (25 mM), cells were subsequently cultured for 24 h in a growth medium supplemented with NH_4_Cl (25 mM). Cells were processed for RT-qPCR **(B)**, immunoblotting **(C, D)**, or plaque assay **(E)**. **(A)** A schematic timeline of the experiment. **(B)** The graph shows the average ± SEM of the Log_10_ transformation of the ratio of NS3 to the mRNA of glyceraldehyde-3-phosphate dehydrogenase (GAPDH, housekeeping gene) as measured by RT-qPCR. Ratios were normalized to the values obtained in untreated conditions (taken as 1). Here, and throughout the figure, significance was calculated by One-Way ANOVA. ***p = 0.0004; ****p < 0.0001. **(C)** Representative immunoblot probed for expression of NS3 and actin (loading control). **(D)** The graph shows the average ± SEM of the Log_10_ transformation of the ratio of NS3 to Actin (housekeeping gene) as measured by densitometry. Ratios were normalized to the values obtained in untreated conditions (taken as 1). *p = 0.0128; ***p < 0.0007. **(E)** The graph shows the average ± SEM of the Log_10_ transformation of the titers of infectious virions as measured by plaque assay. Titers were normalized to the values obtained in untreated conditions (taken as 1). ****p < 0.0001.

Having identified conditions for CME inhibition in MDBK cells, we probed for their effect on EHDV2-Ibaraki infection. Here too, with the objective of focusing on potential effects on early/entry stages of infection, cells were pre-treated (or not) for 30 min with the different compounds, followed by co-incubation of virus ± treatments (1 h), after which cells were washed and incubated exclusively with NH_4_Cl (for 24 h, **Figure 4A**). When assessing NS3 RNA levels by RT-qPCR, the most prominent inhibition was obtained with NH_4_Cl pretreated cells, in accord with the block in inhibition observed in **Supplementary Figure 1**. IKA (at either 0.5 μM or 2 μM) yielded highly significant levels of inhibition, while treatment with dynasore or dynole exhibited a trend towards inhibition of infection, which fell short of significance (**Figure 4B**). Next, we repeated this experiment, with two additional modes of readout: assessment of NS3 protein levels (**Figures 4C, D**), and evaluation of the production of infectious virions (**Figure 4E**). These experiments confirmed the inhibitory potential of NH_4_Cl and IKA while failing to detect significant inhibition of infection by the dynamin inhibitors. Together, these results indicated that inhibition of CME, as observed upon treatment with dynole (as measured by transferrin uptake, **Figure 3**), was insufficient to inhibit EHDV2-Ibaraki entry significantly.

**Figure 5:**
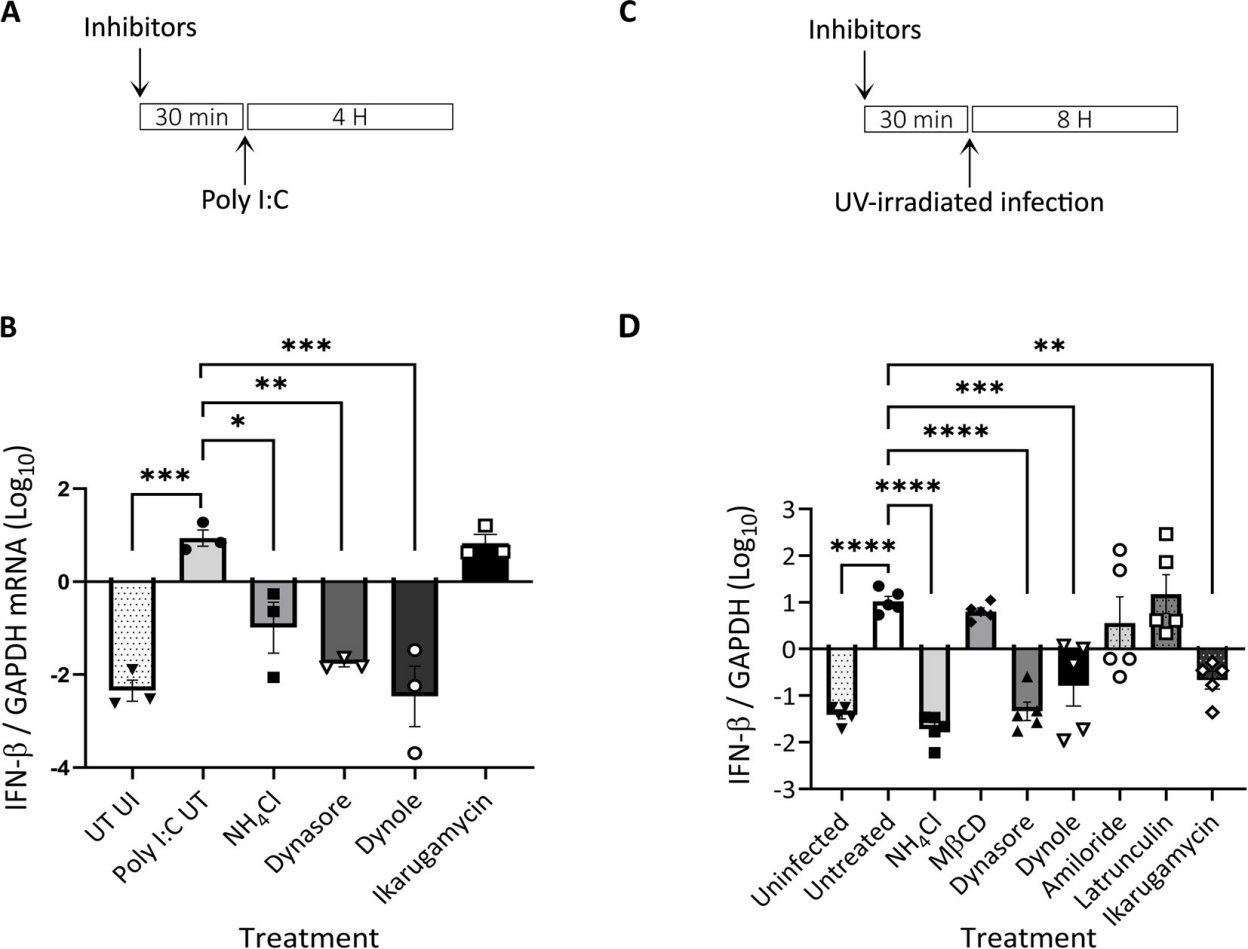
Induction of interferon β (IFN-β) following infection of MDBK with UV-inactivated EHDV2-Ibaraki is inhibited by endosome alkalinization, ikarugamycin or dynamin inhibition. MDBK cells were pre-treated (30 min) with NH_4_Cl (25 mM), methyl-β-cyclodextrin (MβCD, 15 mM), dynasore (80 μM), dynole (40 μM), amiloride (1 mM), latrunculin-B (1 μM), or ikarugamycin (0.5 or 2 μM) prior to incubation with polyI:C [3 µg/ml, 4 h, **(A, B)**] or infection with UV-irradiated EHDV2-Ibaraki [(MOI = 10, 8 h, **(C,D)**] in the same media. Cells were processed for RT-qPCR for measurement of the mRNA levels of interferon-β (IFN-β) or GAPDH. Significance was calculated by One-Way ANOVA. **(A)** A schematic timeline of the polyI:C experiment. **(B)** The graph shows the average ± SEM of the Log_10_ transformation of the ratio of IFN-β to GAPDH mRNA levels as measured by RT-qPCR. **(C)** A schematic timeline of the infection experiment. **(D)** The graph shows the average ± SEM of the Log_10_ transformation of the ratio of IFN-β to GAPDH mRNA levels as measured by RT-qPCR Significance was by One-Way ANOVA. *p<0.05, **p < 0.0025; ***p < 0.001; ****p < 0.0001.

In addition to mediating the initial steps of productive infection of different viruses, the transit through the endocytic/endosomal compartments allows for the sensing of incoming viruses. To assess the sensing of dsRNA, the genetic material of the EHDV2-Ibaraki, and a classical pathogen-associated molecular pattern (PAMP), we opted to measure the level of induction of bovine interferon β (IFN-β) by RT-qPCR. Initially, we stimulated with the dsRNA mimic polyI:C (3 µg⁄ml, 4 h) cells treated or not with NH_4_Cl, dynamin inhibitors (dynasore or dynole), or IKA. This revealed a 3-orders of magnitude increase in IFN-β expression upon polyI:C stimulation, a block in such increase upon dynamin inhibition, a partial decrease in the presence of NH_4_Cl, and no effects of IKA (**Figures 5A, B**). These results suggest a requirement for dynamin activity of sensing of dsRNA, a partial requirement for endosome acidification and a lack of requirement for processes/phenomena regulated by IKA. Next, we repeated the experiment, employing this time UV-inactivated EHDV2-Ibaraki (UV-EHDV2; MOI 10), which is predicted to retain its potential to induce cellular sensing responses to incoming viruses, while being unable to actively infect cells. Initially, we confirmed that UV irradiation inhibited productive EHDV2-Ibaraki infection, as shown by a complete lack of NS3 expression in cells infected with UV-EHDV2 (**Supplementary Figure 6**). Subsequently, we treated MDBK cells as in **Figure 4** or **Supplementary Figure 2**, and stimulated them with UV-EHDV2 (MOI=10, 8h). Here, too, untreated cell stimulation resulted in increased IFN-β expression by more than 3 orders of magnitude (**Figures 5C, D**). NH_4_Cl treatment abrogated the virus-mediated increase, indicating the requirement for endosomal pH for the viral sensing. Notably, dynasore inhibited IFN-β upregulation similarly to NH_4_Cl, suggesting a strict requirement for functional dynamin-dependent pathways. While MβCD, latrunculin, or amiloride were devoid of inhibitory effects, IKA and dynole significantly inhibited IFN-β expression, albeit less efficiently than dynasore (**Figure 5D**). Together, these results support the notion that dynamin activity is required for dsRNA sensing in both forms of stimuli, polyI:C and inactivated viruses. The greater potency of inhibition observed for NH_4_Cl and IKA in the case of UV-EHDV2 (relative to polyI:C) suggests an impediment to the processing of incoming viruses, which may be required for its sensing.

### 3.4 IKA alters the morphology of internal membrane compartments

**Figure 6:**
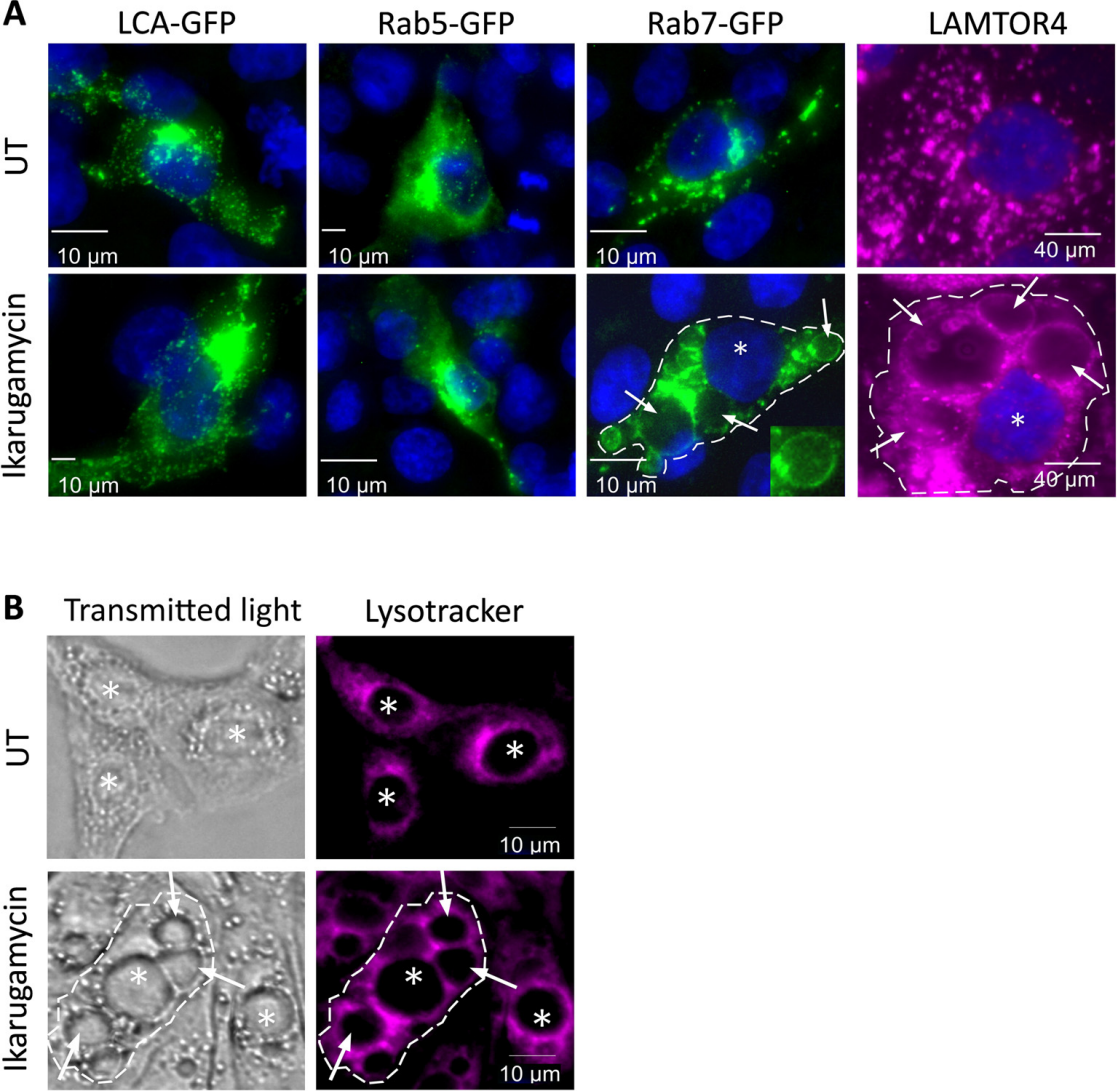
Ikarugamycin induces vacuolation and alters the intracellular distributions of Rab7 and LAMTOR4. **(A)** MDBK cells were transfected or not with clathrin-light chain A fused to GFP (LCA-GFP; first column), Rab5 fused to GFP (Rab5-GFP, second) or Rab7 fused to GFP (Rab7-GFP, third column). For these columns, at 12 h post-transfection, cells were treated (or not) with IKA (1 μM, 24 h). Following fixation-permeabilization, cells were stained with DAPI for visualization of nuclei and imaged by fluorescence microscopy. For the fourth column, untransfected cells were treated or not with IKA (1 µM, 24 h). Following fixation, the cells were consecutively stained against LAMTOR4, followed by DAPI staining. In the third and fourth columns, the dashed line delimits the boundaries of one cell in the field, the star (*) represents the nucleus, and the arrows point at the enlarged vacuoles induced by IKA. Micrographs depict typical fields of the different transfection/treatment conditions. Bars represent 10μm. **(B)** MDBK cells were pre-treated (or not) with IKA (2 μM, 24 h). Cells were subsequently incubated with lysotracker (30 μM, 25 min) and taken for live imaging. Lysotracker accumulates within acidic compartments (red). The dashed line, stars (*), arrows and bars are as in **(A)**.

Given that the viral entry process integrates initial events at the plasma membrane with delivery and processing in endosomes, we hypothesized that part of the inhibitory effects of IKA on productive infection or sensing may stem from effects downstream of internalization. This hypothesis was strongly supported by the extensive vacuolation, in cells treated with IKA (1 µM, 20 h, quantified in **Supplementary Figure 7**). Next, we probed for effects of IKA on the intracellular distribution of clathrin light chain A-GFP (LCA-GFP, a marker of clathrin-coated pits and vesicles and clathrin-coated endosomes), Rab5-GFP (a marker of early endosomes) or Rab7-GFP (a marker of late endosomes). While no apparent IKA-induced modifications to the intracellular distribution of either LCA-GFP or Rab5-GFP were observed (**Figure 6A**), a marked portion of Rab7-GFP-expressing cells exhibited localization of this protein to the boundaries of vacuole-like structures upon IKA treatment (**Figure 6A**). This was further confirmed via staining of IKA-treated (1 µM, 20 h) or naïve cells against LAMTOR4, an endogenous marker of late endosomes/lysosomes. This revealed, marked vacuolization in IKA-treated cells (**Figure 6A**). To further characterize the vacuoles, we fed MDBK cells (treated or not with IKA) with lysotracker. This revealed typical perinuclear staining in untreated cells and a lack of accumulation in the IKA-induced vacuoles (**Figure 6B**), suggesting that either the vacuoles are not sufficiently acidic to entrap lysotracker or that they are inaccessible to this agent.

**Figure 7:**
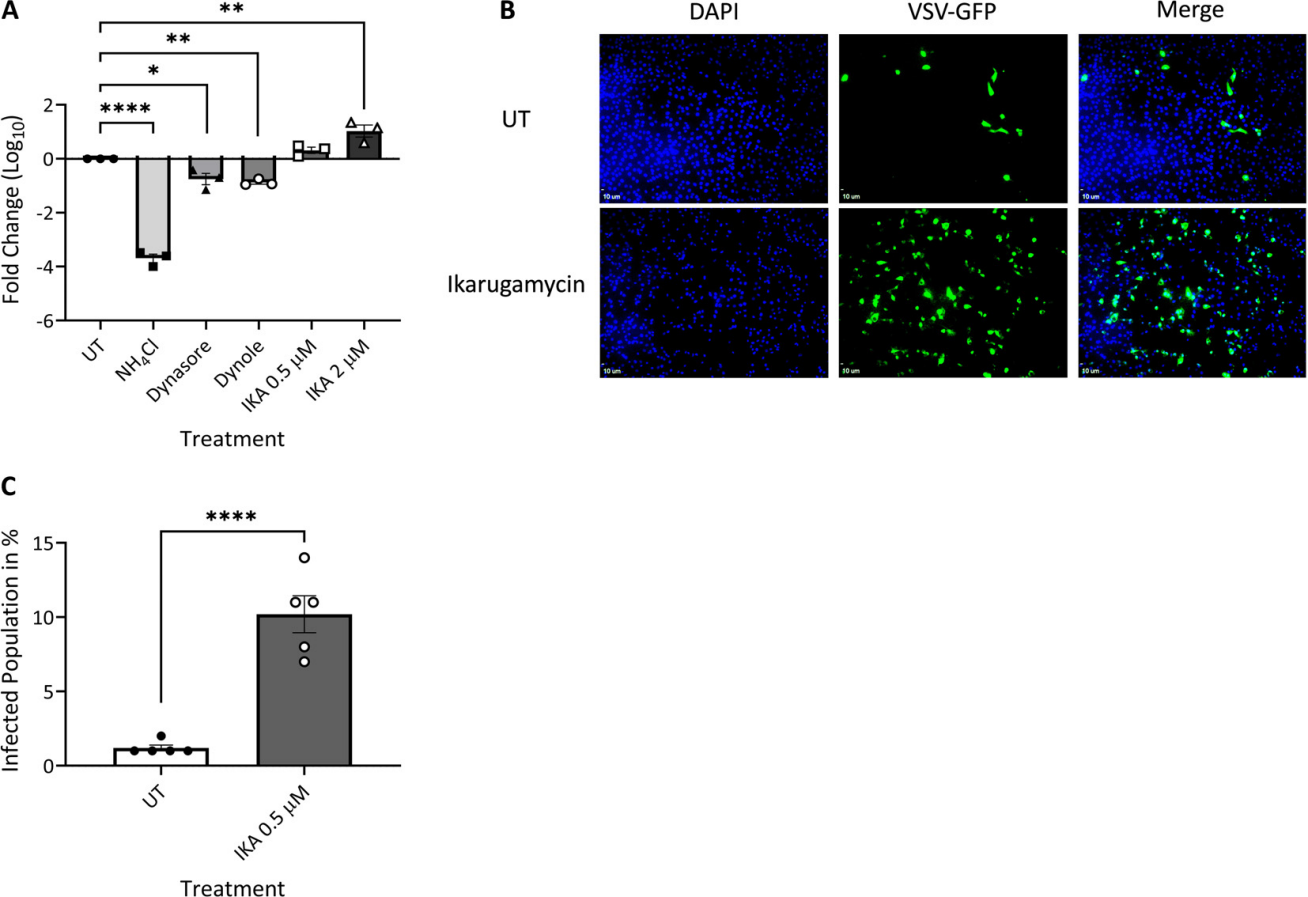
Ikarugamycin enhances VSV infection. MDBK cells were pre-treated (30 min) with NH_4_Cl (25 mM), dynasore (80 μM), dynole (40 μM), or Ikarugamycin (IKA, 0.5 or 2 μM), followed by infection with VSV (MOI = 1, 1 h, in the presence of the inhibitors). Subsequently, cells were washed with PBS + NH_4_Cl (25 mM) and cultured in a growth medium containing NH_4_Cl (25 mM) for 24 h. **(A)** Titers were normalized to the values obtained in untreated conditions (UT, taken as 1). The graph shows the average ± SEM of the Log_10_ transformation of the ratio of titers of infectious virions (condition/untreated (UT) as measured by plaque assay. *p = 0.0169; **p < 0.007 ; ****p < 0.0001. **(B, C)** MDBK cells were pre-treated and treated or not with IKA (0.5 μM) as described in **(A)** and infected with a VSV virus encoding for GFP (VSV-GFP). Following fixation-permeabilization, cells were stained with DAPI and imaged with a fluorescence microscope. **(B)** Fluorescence micrographs of typical fields. Bars represent 10 μm. **(C)** The graph shows the average ± SEM of the percentage of infected cells in the different conditions. Significance was calculated by an unpaired two-tailed t-test. ****p < 0.0001.

To further probe for the effects of NH_4_Cl, dynasore, dynole, or IKA on the entry of different viruses, we assessed VSV or BTV-8 infection of MDBK cells, under analogous conditions to those employed for the above-described experiments with EHDV2-Ibaraki. Multiple studies (Johannsdottir et al., 2009; Cabot et al., 2022; Roche et al., 2008; Cureton et al., 2009, 2010) have established the dependency of VSV entry on clathrin-mediated endocytosis and endosomal acidification. Per the latter requirement, NH_4_Cl abrogated VSV infection as measured by the marked reduction in the production of infectious virions at 24 h (**Figure 7A**). Dynasore and dynole resulted in partial but significant inhibition of VSV infection, confirming the role of dynamin-dependent processes (e.g. CME) in VSV entry. Surprisingly, IKA not only failed to inhibit VSV infection/entry but rather enhanced the production of infectious virions in a concentration-dependent manner (**Figure 7A**). This effect was further confirmed through assessment of the percentage of cells (treated or not with IKA) exhibiting GFP expression following infection with a GFP-expressing VSV clone (**Figures 7B, C**). These results suggest either an IKA-mediated enhancement of an alternative entry pathway for VSV or an enhancement of infection due to IKA-mediated alterations to the endosomal compartments. In contrast, infection of MDBK cells with BTV-8, which is expected to exhibit very similar endosomal-processing requirements as EHDV2-Ibaraki, was inhibited by NH_4_Cl and IKA (**Supplementary Figure 8**). Together, our results reinforce the prominence of endosomal processing in regulating the early steps of infection of EHDV2-Ibaraki, BTV-8, and VSV.

## 4 Discussion

### 4.1 Alkalinization-mediated inhibition of EHDV2-Ibaraki infection and sensing

As per our results, EHDV2-Ibaraki, VSV, and BTV-8 depend on acidic compartments within the endosomal-lysosomal system of the cell for productive infection of bovine cells, as demonstrated by the robust inhibition in infection observed in cells treated with NH_4_Cl before and throughout infection. The requirement for luminal acidic pH for VSV or BTV entry was previously reported (Hyatt et al., 1989; Forzan et al., 2007; Beilstein et al., 2020; Cabot et al., 2022). As measured for EHDV2-Ibaraki, the alkalinization-mediated inhibition of infection was dependent on the time of addition of NH_4_Cl, suggesting the pH dependence of early but not late stages of infection. This time dependence of the NH_4_Cl allowed us to measure the entry window of EHDV2-Ibaraki, which completed its passage through low pH compartments within 3h, with a half-time of ~ 90 min. The NH_4_Cl-mediated block in IFN-β induction, which is expected to occur upon revealing EHDV2-Ibaraki dsRNA, further supports the notion of the alkalinization-mediated interference to viral processing and/or viral degradation. However, NH_4_Cl also partially inhibited IFN-β induction by polyI:C. This partial inhibition may reflect the requirement for acidic pH for toll-like receptor 3 to recognize dsRNA and activate downstream signaling (de Bouteiller et al., 2005). A partial reduction in transferrin accumulation in MDBK cells was also induced by NH_4_Cl, suggesting that part of its effects may stem from interference with trafficking steps. Indeed, inhibition of endocytosis was observed upon NH_4_Cl treatment of mouse tubular cells, possibly reflecting the pH-sensitive recruitment of trafficking regulators to endosomal membranes (Hurtado-Lorenzo et al., 2006; Aniento et al., 1996). However, the differences in the magnitude of the near-complete block in infection as opposed to the minor reduction in transferrin accumulation led us to conclude that the main effect of NH_4_Cl reflects the requirement of endosomal pH for viral processing.

### 4.2 Inhibition of individual endocytic pathways or mediators fails to block EHDV2-Ibaraki infection

Inhibition of dynamin-, actin- or cholesterol-mediated endocytosis with dynasore/dynole, latrunculin, or methyl-β-cyclodextrin, respectively; or macropinocytosis with amiloride; all failed to significantly inhibit EHDV2-Ibaraki infection of MDBK (as measured by expression of viral mRNA, viral protein or production of infectious virions). The inhibition of dynamin is expected to affect the entirety of CME and a subset of the clathrin-independent endocytic pathways that depend on dynamin for vesicle internalization (Antonny et al., 2016; Macia et al., 2006). Accordingly, transferrin uptake (**Figure 3**) and VSV infection were significantly inhibited by the dynole or dynasore, both of which inhibit the GTPase activity of dynamins (Macia et al., 2006; Hill et al., 2009). Cholesterol depletion is predicted to affect non-clathrin (e.g., caveolin-mediated endocytosis) and at least a subset of clathrin-mediated endocytic events, leading to the membrane retention of ligands that employ multiple entry pathways such as cholera toxin (Massol et al., 2004; Torgersen et al., 2001). Moreover, actin dynamics, which are inhibited by latrunculin (Spector et al., 1983) were proposed to be involved in several entry pathways, including a subset of clathrin-mediated events (Yarar et al., 2005; Boucrot et al., 2006; Cureton et al., 2009), fast-endophilin-mediated-endocytosis [FEME (Watanabe and Boucrot, 2017)] and entry mediated by clathrin and dynamin-independent carriers which form GPI-enriched endocytic compartments [CLIC/GEEC (Sathe et al., 2018)] pathways; in addition to being required for macropinocytosis (Salloum et al., 2023; Mylvaganam et al., 2021). The latter pathway is also predicted to be inhibited by amiloride via the acidification of the cytosol (Koivusalo et al., 2010). Thus, the lack of inhibition of EHDV2-Ibaraki infection by any of these treatments supports the notion that inhibiting any single endocytic pathway or inhibiting a selection of entry pathways does not suffice for blocking EHDV2-Ibaraki entry into MDBK cells. If the amount of virus entering through a specific pathway was constant (i.e., equal in cells treated or not with an inhibitor of a different pathway), one would expect that inhibition of this pathway would result in a proportional reduction in productive viral infection relative to the percentage of viruses entering via this specific pathway. However, this is not observed, as inhibition of specific pathways (or of pathways that depend on common requirements such as actin dynamics, dynamin activity, or membrane cholesterol content) during the window of entry of EHDV2-Ibaraki did not significantly reduce viral infection. Two possible explanations for this phenomenon are that either the virus enters through a pathway insensitive to all treatments employed in this study or cells can compensate for inhibiting certain pathways through the upregulation of others, which EHDV2-Ibaraki may also employ for entry. We favor the latter scenario, as such compensation has been reported [e.g., in the upregulation of clathrin-independent pinocytosis upon inhibition of dynamin (Damke et al., 1995)]. Similar to our results, influenza was shown to employ clathrin-coated vesicles as a non-exclusive mode of entry into cells (Bao et al., 2021; Lakadamyali et al., 2004; Mazel-Sanchez et al., 2023; Rust et al., 2004; Sun and Whittaker, 2013), but inhibition of clathrin-mediated endocytosis via expression of the Δ95-295 mutant of Eps15 or chlorpromazine failed to inhibit influenza infection in HeLa cells (Sieczkarski and Whittaker, 2002). Entry via multiple endocytic pathways may be a common characteristic of viruses that employ oligosaccharides as the element for cell binding and entry, such as influenza (Sun and Whittaker, 2013), as the oligosaccharides may be attached to receptors endowed with different endocytic determinants. Of note, BTV (which exhibits high similarity to EHDV) employs sialic acid to bind and enter cells (Wu and Roy, 2022; Zhang et al., 2010) and was reported to enter either via clathrin or macropinocytosis with dependence on cell type (Forzan et al., 2007; Gold et al., 2010; Stevens et al., 2019).

### 4.3 Ikarugamycin inhibits CME, modifies the endosomal compartments, and interferes with the early steps of EHDV2-Ibaraki infection

An apparent discrepancy is revealed when comparing the effects of treatments within the NH_4_Cl-sensitive window with dynamin inhibitors or IKA on EHDV2-Ibaraki infection. While both inhibit the CME-dependent accumulation of transferrin (particularly notable for dynole and IKA), only IKA inhibits EHDV2-Ibaraki infection. A plausible explanation for this is that CME inhibition does not suffice to block EHDV2-Ibaraki infection, and IKA’s ability to do so depends on other effects of this compound. Indeed, cells treated with IKA exhibited a prominent accumulation of vacuole-like structures, decorated with Rab7 or LAMTOR4 and which failed to accumulate lysotracker in their interior. This suggests that such structural (and likely function) modification of Rab7/LAMTOR4 compartments may have functional implications for EHDV2-Ibaraki infection. Interestingly, a reverse discrepancy was observed for VSV. Here, dynamin inhibitors significantly reduced infection, while IKA increased infection by an order of magnitude. This lack of inhibition of VSV by IKA suggests that in MDBK cells VSV may employ clathrin-independent carriers for entry and employ distinct endosomal compartments as compared with EHDV2-Ibaraki (or BTV-8). In line with this putative difference in the endosomal compartment employed for entry, the pH requirements for viral entry were reported to differ between VSV [~6.2 pH (Johannsdottir et al., 2009)] and BTV [~5.5 pH (Wu et al., 2019)]. Of note, IKA was shown to affect autophagic flux and induce TFEB signaling (Wang et al., 2017), which may be related to its effects on late endosomes. One feature of IKA that may endow it with the ability to alter endosomes is its strong affinity for sodium ions (Paquette et al., 1990), which are required for pH regulation of endosomes/lysosomes (Steinberg et al., 2010; Chadwick et al., 2021; Scott and Gruenberg, 2011). Notably, conditions that induce similar cell vacuolation, such as treatment with the phosphatidylinositol-3-phosphate 5-kinase inhibitor apilimod or deficient expression of VPS29, also inhibited viral infection (Kreutzberger et al., 2021; Nelson et al., 2017; Poston et al., 2022). The case of VPS29 deficiency is of particular interest as there too the effects were virus-specific, causing inhibition of HCoV-OC43 or SARS-CoV-2, and facilitation of influenza infection (Poston et al., 2022). Thus, while the hampering of endosome functions is an attractive target for antiviral therapies, the generality of such approaches towards different viruses may be restricted by the heterogeneity in endosomal requirements of such viruses.

## Data Availability

The raw data supporting the conclusions of this article will be made available by the authors, without undue reservation.

## Supplementary material

Supplementary Figure 1 Alkalinization of endosomal compartments with NH_4_Cl blocks EHDV2-Ibaraki infection of MDBK cells. MDBK cells, plated on glass coverslips, were left untreated/uninfected, infected with EHDV2-Ibaraki in the normal growth medium (MOI=1, 24 h), or infected in the presence of NH_4_Cl (25 mM, added 30 min before addition of virus, MOI=1, 24 h). Cells were fixed and stained against the non-structural protein 3 of EHDV2-Ibaraki (NS3, green) or with phalloidin (red) or DAPI (blue). Fluorescence micrographs depict typical fields.

Supplementary Figure 2 Cholesterol depletion, actin polymerization inhibition, or macropinocytosis inhibition do not affect the early steps of EHDV2-Ibaraki infection. MDBK cells were pre-treated or not with NH_4_Cl (25 mM), methyl-β-cyclodextrin (MβCD, 15 mM), amiloride (1 mM), or latrunculin-B (1 μM) for 30 min, followed by infection with EHDV2-Ibaraki (MOI = 1, 1 h) in the presence of the inhibitors (except for untreated sample). Following extensive washes with PBS supplemented with NH_4_Cl (25 mM), cells were cultured in a growth medium supplemented with NH_4_Cl (25 mM, 24 h) to block further entry. **(A)** A schematic timeline of the experiment. **(B)** The graph shows the average ± SEM of the Log_10_ transformation of the ratio of NS3 to Actin (housekeeping gene) as measured by densitometry. Ratios were normalized to the values obtained in untreated conditions (taken as 1). **(C)** The graph shows the average ± SEM of the Log_10_ transformation of the titers of infectious virions as measured by plaque assay. Titers were normalized to the values obtained in untreated conditions (taken as 1). Significance was calculated by One-Way ANOVA. ****, p < 0.0001.

Supplementary Figure 3 Latrunculin control. MDBK cells were treated (or not) with latrunculin-B (1 μM) for 30 or 90 minutes. Following fixation-permeabilization, cells were stained with DAPI (blue) and phalloidin (red) and imaged by fluorescence microscopy. Bars correspond to 10 μm.

Supplementary Figure 4 Ikarugamycin blocks transferrin internalization. MDBK cells, in serum-free medium, were left untreated or pre-treated with IKA (0.5 or 2 μM, 30 min) followed by incubation with transferrin (50 μg/ml, 20 min) in the same medium. Before fixation, cells were washed with acid wash buffer (150 mM NaCl, 0.1 M Glycine, pH 2.5) to eliminate membrane-localized transferrin. Following fixation-permeabilization, cells were stained with DAPI and imaged with a fluorescence microscope. **(A)** Micrographs depict two-dimensional projections of volumetric imaging of typical fields. DAPI (blue), transferrin (green). Bars represent 10μm. **(B)** The graph shows the average ± SEM of the ratio of the transferrin signal divided by DAPI signal. Calculation of statistical significance was carried out with a two-tailed t-test. ***, p = 0.0009, *, p = 0.0305.

Supplementary Figure 5 Ikarugamycin blocks the internalization of the type II transforming growth factor-β receptor (TβRII). COS7 cells were transfected with a plasmid encoding for an extracellularly Myc-epitope-tagged construct of TβRII. 24 h after transfection, cells were left untreated or treated with Ikarugamycin (B, 2 µM, 30 min) and subsequently labeled in the cold with anti-Myc antibodies. Samples were then to 37°C degrees for the indicated times to allow for internalization in the same medium as the pre-treatment. **(A)** Cells were treated or not with acid-wash buffer (150 mM NaCl, 0.1 M Glycine, pH 2.5) to dissociate antibodies bound to plasma-membrane-localized Myc-TβRII. **(B)** Cells were pre-treated/treated or not with 2 µM ikarugamycin.

Supplementary Figure 6 UV-irradiated EHDV2-Ibaraki does not productively infect MDBK cells. MDBK cells were infected (or not) with EHDV2-Ibaraki or UV-radiated EHDV2-Ibaraki for 24 h. **(A)** Representative immunoblot of the EHDV2-Ibaraki non-structural protein 3 (NS3) and Actin (loading control). **(B)** The graph shows the average ± SEM of the ratio of NS3 to actin signals as measured by densitometry. Significance was calculated with One-way ANOVA. ****, p < 0.0001.

Supplementary Figure 7 Ikarugamycin induces the vacuolization of MDBK cells. MDBK cells, grown on coverslips were left untreated or treated with ikarugamycin (1 µM, 24 h). Cells were then fixed, permeabilized, and stained with DAPI (blue) or against LAMTOR4 (pink). **(A)** Typical field of cells as imaged by fluorescence microscopy. Bars correspond to 40 µm. **(B)** The graph shows the average ± SEM of the percentage of cells exhibiting enlarged vacuoles, as determined by visual inspection of multiple fields (n=6 for each condition).

Supplementary Figure 8 Endosome alkalinization and ikarugamycin inhibit BTV-8 infection of MDBK cells. BTV cells were left untreated or pre-treated with NH_4_Cl (25 mM, 30 min) or ikarugamycin (0.5, 1 or 2 µM, 30 min) before infection with BTV-8 (MOI = 0.1, 1 h, in the same medium of pre-treatment). Following extensive washes with PBS supplemented with 25 mM NH_4_Cl, cells were incubated with growth medium supplemented with 25 mM NH_4_Cl for an additional 24 h. Subsequently, samples were processed for RT-PCR. **(A)** Representative DNA electrophoresis of RT-PCR products at 25 PCR amplification cycles. Samples are marked as: untreated-uninfected, UT/UI; untreated-infected, UT/INF; NH_4_Cl-treated-infected, NH_4_Cl/INF; ikarugamycin-treated-infected, IKA/INF (at three concentrations: 0.5 µM, 1 µM or 2 µM). **(B)** The graph shows the average ± SEM of the ratio of signals obtained for BTV8 divided the signals obtained for Actin (housekeeping gene) as measured by RT-qPCR.
