# Supplementary material for: Inhibition of early EHDV2-Ibaraki infection steps in bovine cells by endosome alkalinization or ikarugamycin, but not by blockage of individual endocytic pathways
Source: Front Cell Infect Microbiol. 2025 Feb 6;15:1494200. doi: 10.3389/fcimb.2025.1494200 (PMC11839642; doi:10.3389/fcimb.2025.1494200)
Supplement: Supplementary Figure 1 — Alkalinization of endosomal compartments with NH4Cl blocks EHDV2-Ibaraki infection of MDBK cells. MDBK cells, plated on glass coverslips, were left untreated/uninfected, infected with EHDV2-Ibaraki in the normal growth medium (MOI=1, 24 h), or infected in the presence of NH4Cl (25 mM, added 30 min before addition of virus, MOI=1, 24 h). Cells were fixed and stained against the non-structural protein 3 of EHDV2-Ibaraki (NS3, green) or with phalloidin (red) or DAPI (blue). Fluorescence micrographs depict typical fields. [file DataSheet1.pdf]

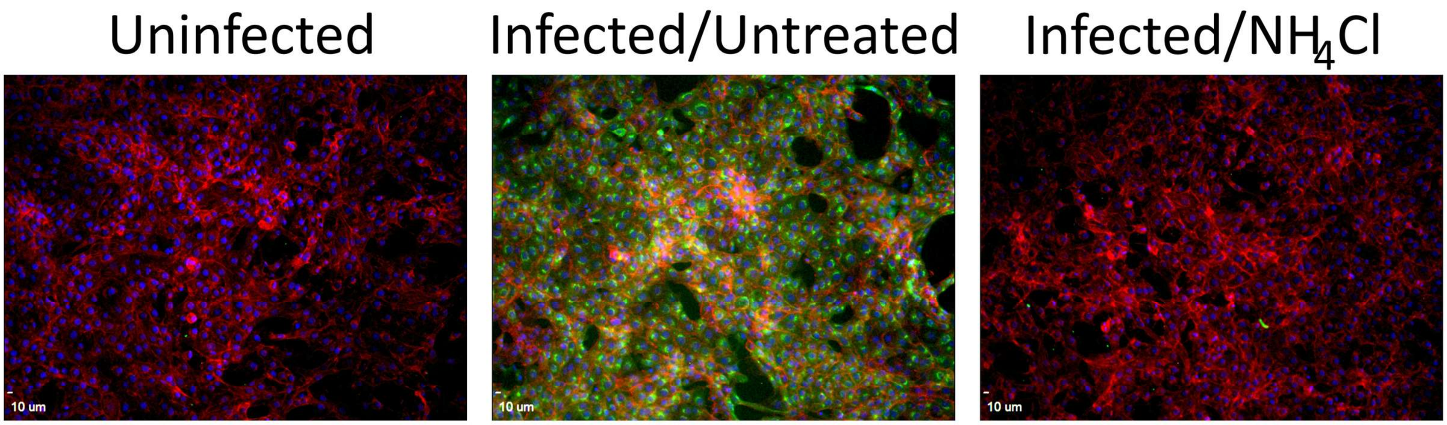

Sup. Figure 1 | Alkalinization of endosomal compartments with NH4Cl blocks EHDV2-Ibaraki infection of MDBK cells. MDBK cells, plated on glass coverslips, were left untreated/uninfected, infected with EHDV2-Ibaraki in the normal growth medium (MOI=1, 24 h), or infected in the presence of NH4Cl (25 mM, added 30 min before addition of virus, MOI=1, 24 h). Cells were fixed and stained against the non-structural protein 3 of EHDV2-Ibaraki (NS3, green) or with phalloidin (red) or DAPI (blue). Fluorescence micrographs depict typical fields.

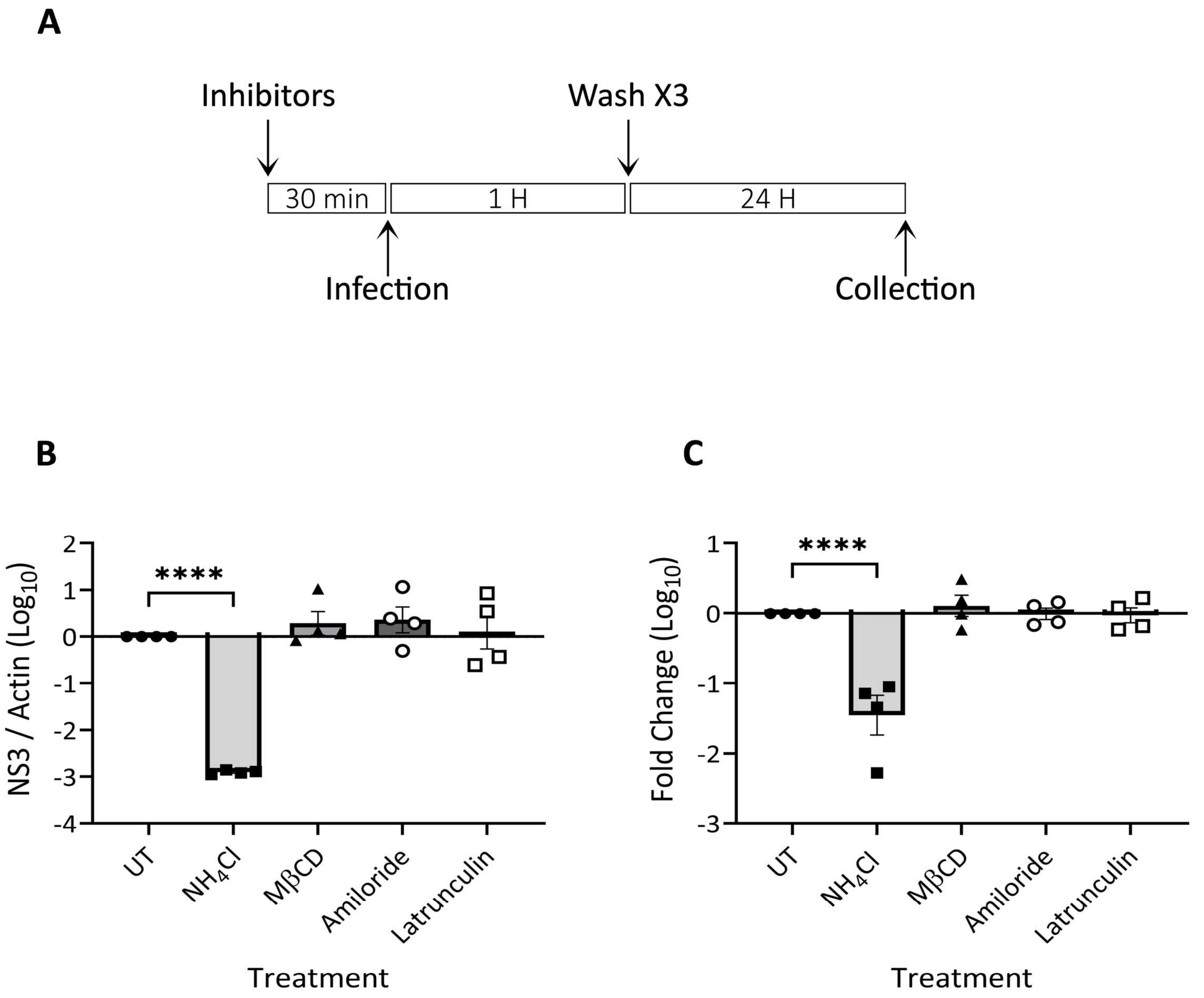

Sup. Figure 2 | Cholesterol depletion, actin polymerization inhibition, or macropinocytosis inhibition do not affect the early steps of EHDV2-Ibaraki infection. MDBK cells were pre-treated or not with NH4Cl (25 mM), methyl-β-cyclodextrin (MβCD, 15 mM), amiloride (1 mM), or latrunculin-B (1 µM) for 30 min, followed by infection with EHDV2-Ibaraki (MOI = 1, 1 h) in the presence of the inhibitors (except for untreated sample). Following extensive washes with PBS supplemented with NH4Cl (25 mM), cells were cultured in a growth medium supplemented with NH4Cl (25 mM, 24 h) to block further entry. (A) A schematic timeline of the experiment. (B) The graph shows the average ± SEM of the Log10 transformation of the ratio of NS3 to Actin (housekeeping gene) as measured by densitometry. Ratios were normalized to the values obtained in untreated conditions (taken as 1). (C) The graph shows the average ± SEM of the Log10 transformation of the titers of infectious virions as measured by plaque assay. Titers were normalized to the values obtained in untreated conditions (taken as 1). Significance was calculated by One-Way ANOVA. ****, $p < 0.0001$.

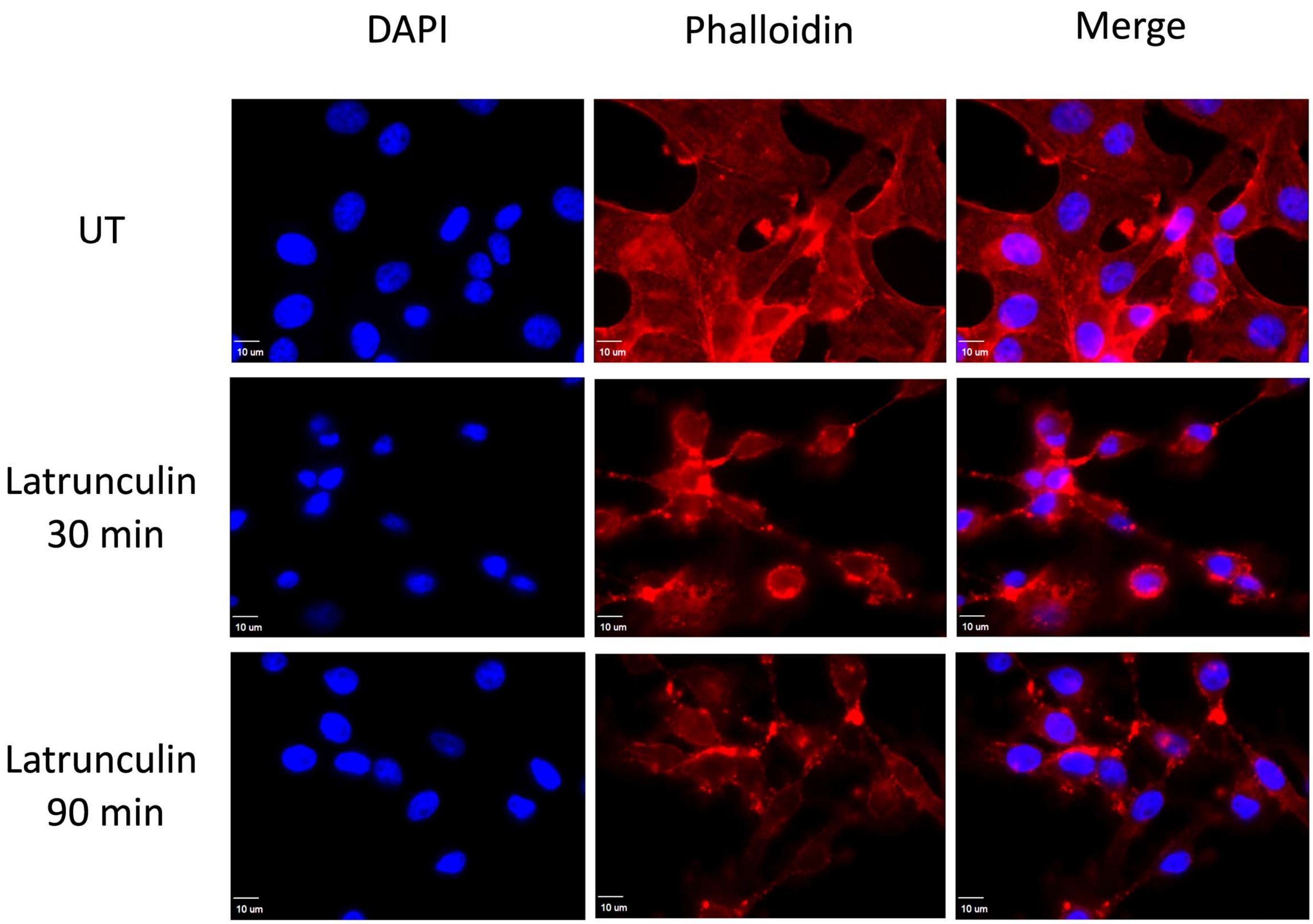

Sup. Figure 3 | Latrunculin control. MDBK cells were treated (or not) with latrunculin-B (1 µM) for 30 or 90 minutes. Following fixation-permeabilization, cells were stained with DAPI (blue) and phalloidin (red) and imaged by fluorescence microscopy. Bars correspond to 10 µm.

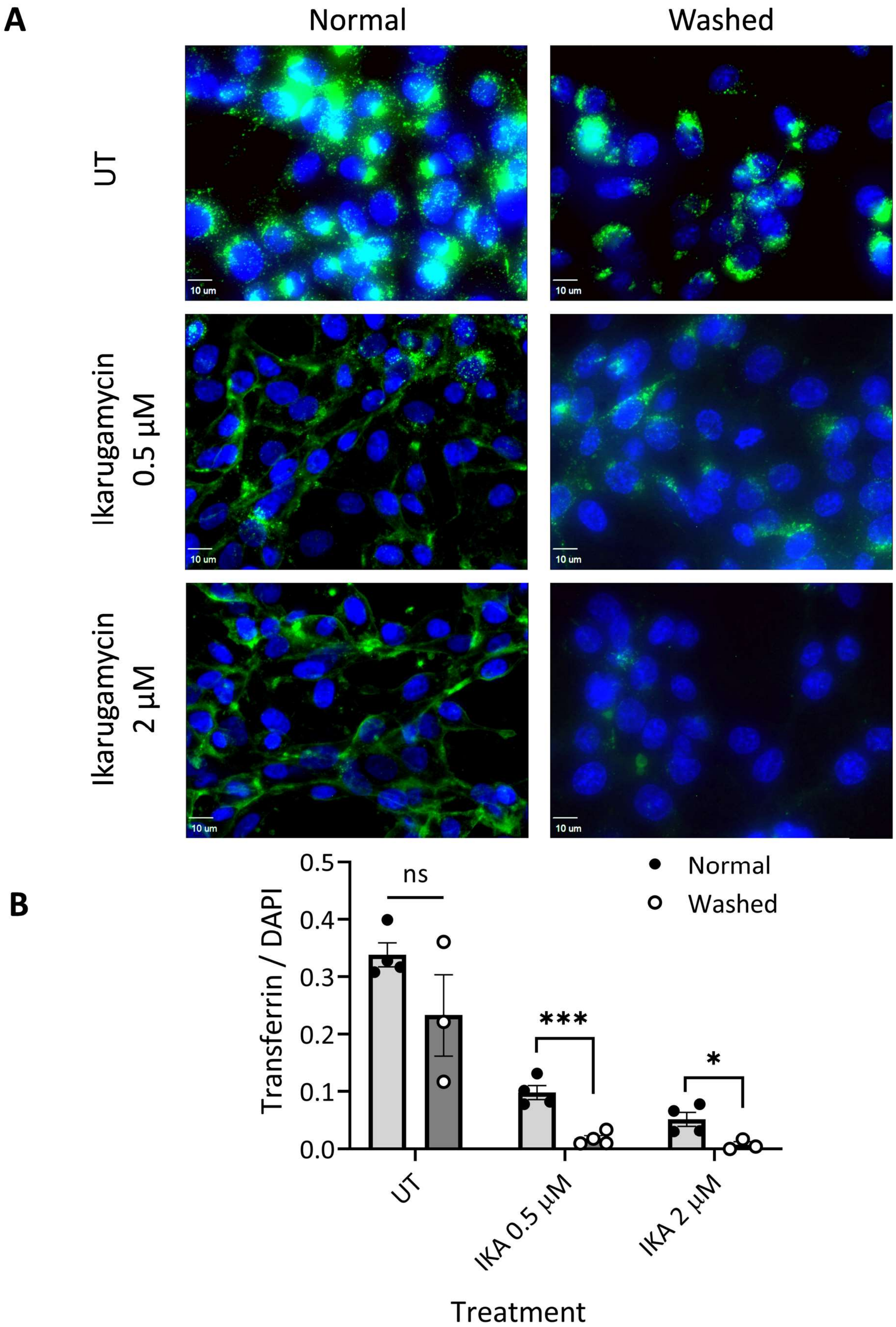

Sup. Figure 4 | Ikarugamycin blocks transferrin internalization. MDBK cells, in serum-free medium, were left untreated or pre-treated with IKA (0.5 or 2 µM, 30 min) followed by incubation with transferrin (50 µg/ml, 20 min) in the same medium. Before fixation, cells were washed with acid wash buffer (150 mM NaCl, 0.1 M Glycine, pH 2.5) to eliminate membrane-localized transferrin. Following fixation-permeabilization, cells were stained with DAPI and imaged with a fluorescence microscope. (A) Micrographs depict two-dimensional projections of volumetric imaging of typical fields. DAPI (blue), transferrin (green). Bars represent 10µm. (B) The graph shows the average ± SEM of the ratio of the transferrin signal divided by DAPI signal. Calculation of statistical significance was carried out with a two-tailed t-test. ***, $p = 0.0009$, *, $p = 0.0305$.

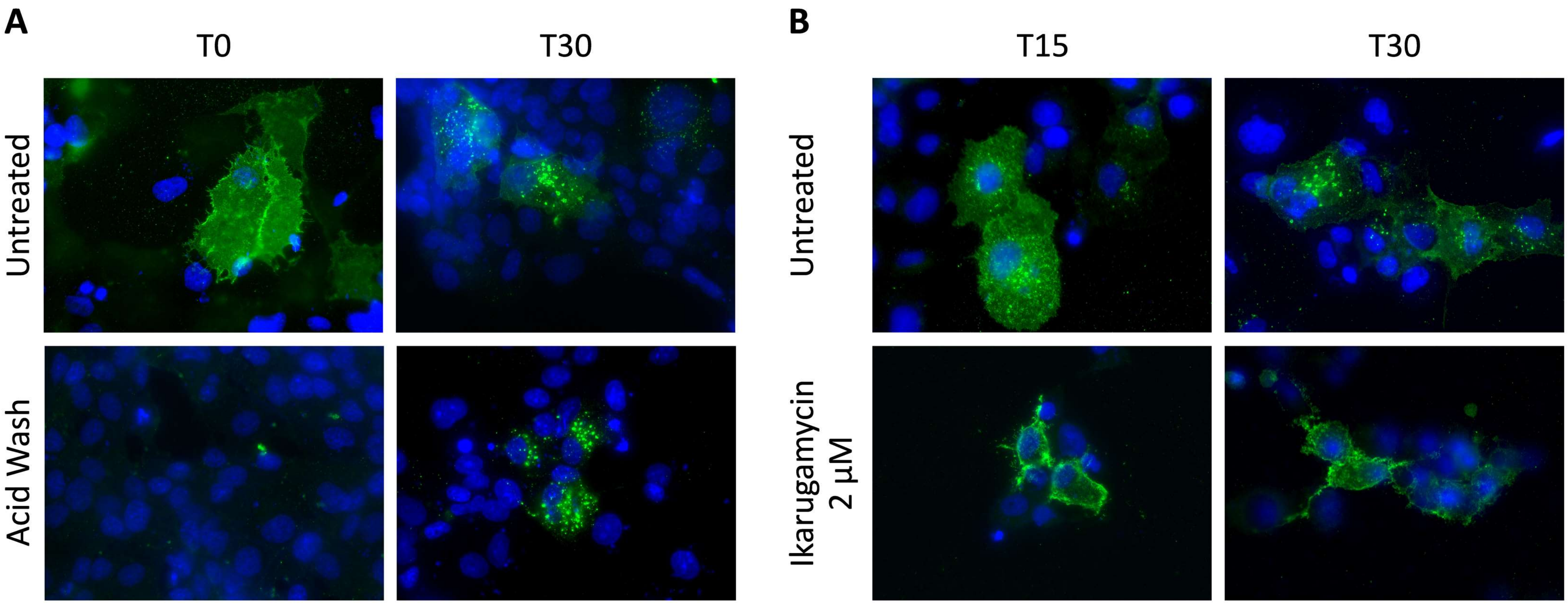

Sup. Figure 5 | Ikarugamycin blocks the internalization of the type II transforming growth factor-β receptor (TβRII). COS7 cells were transfected with a plasmid encoding for an extracellularly Myc-epitope-tagged construct of TβRII. 24 h after transfection, cells were left untreated or treated with Ikarugamycin (B, 2 µM, 30 min) and subsequently labeled in the cold with anti-Myc antibodies. Samples were then to 37 °C degrees for the indicated times to allow for internalization in the same medium as the pre-treatment. (A) Cells were treated or not with acid-wash buffer (150 mM NaCl, 0.1 M Glycine, pH 2.5) to dissociate antibodies bound to plasma-membrane-localized Myc-TβRII. (B) Cells were pre-treated/treated or not with 2 µM ikarugamycin.

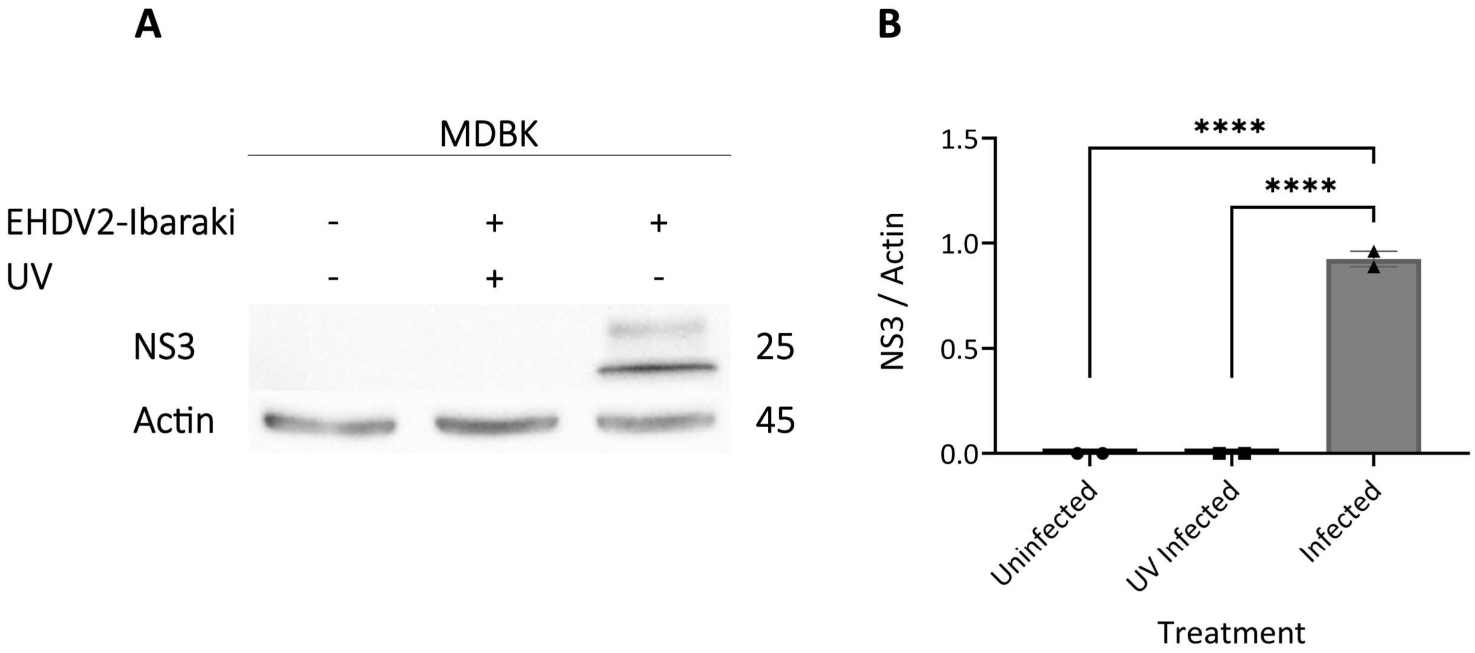

Sup. Figure 6 | UV-irradiated EHDV2-Ibaraki does not productively infect MDBK cells. MDBK cells were infected (or not) with EHDV2-Ibaraki or UV-radiated EHDV2-Ibaraki for 24 h. (A) Representative immunoblot of the EHDV2-Ibaraki non-structural protein 3 (NS3) and Actin (loading control). (B) The graph shows the average ± SEM of the ratio of NS3 to actin signals as measured by densitometry. Significance was calculated with One-way ANOVA. ****, $p < 0.0001$.

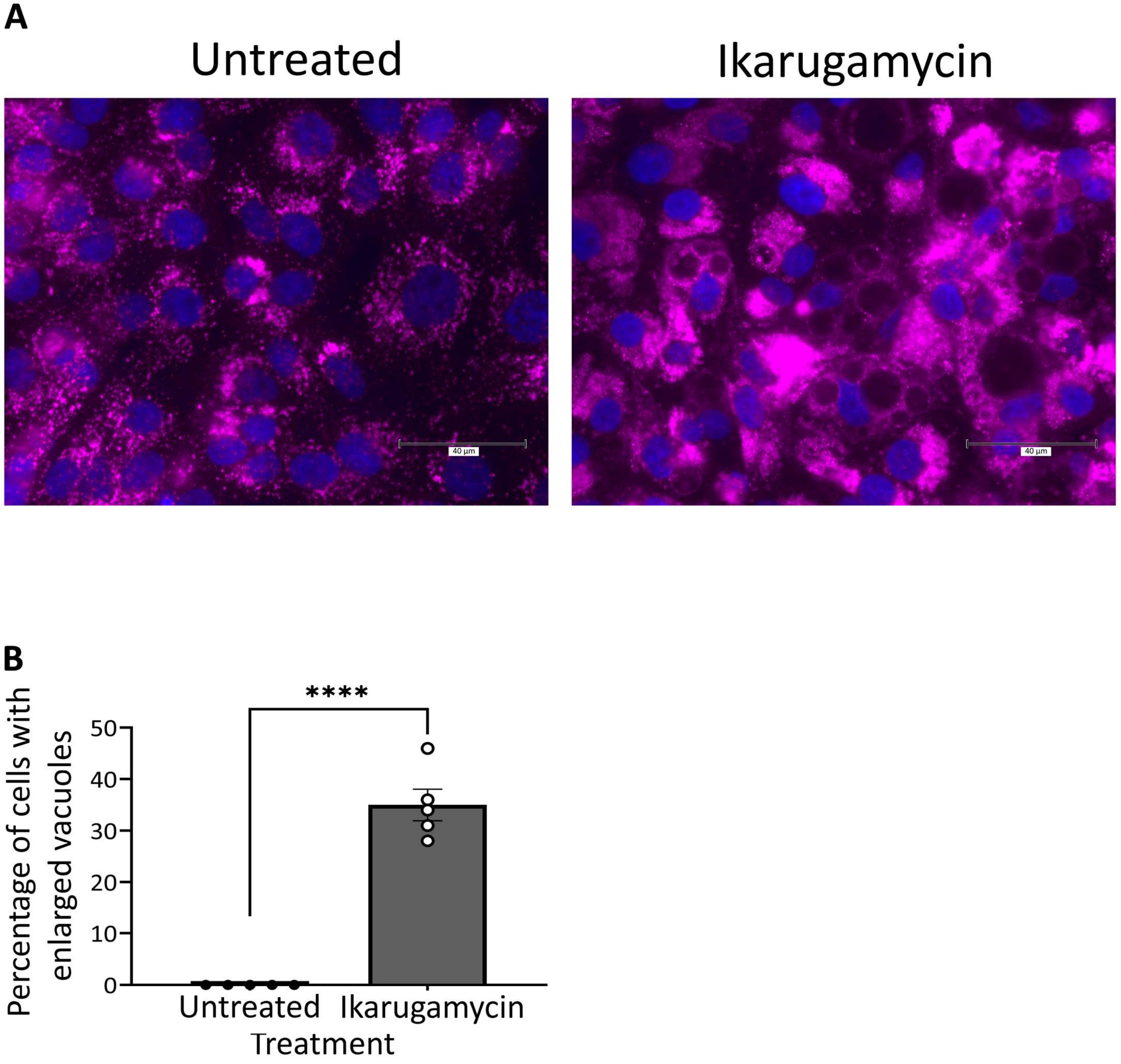

Sup. Figure 7 | Ikarugamycin induces the vacuolization of MDBK cells. MDBK cells, grown on coverslips were left untreated or treated with ikarugamycin (1 µM, 24 h). Cells were then fixed, permeabilized, and stained with DAPI (blue) or against LAMTOR4 (pink). (A) Typical field of cells as imaged by fluorescence microscopy. Bars correspond to 40 µm. (B) The graph shows the average ± SEM of the percentage of cells exhibiting enlarged vacuoles, as determined by visual inspection of multiple fields (n=6 for each condition).

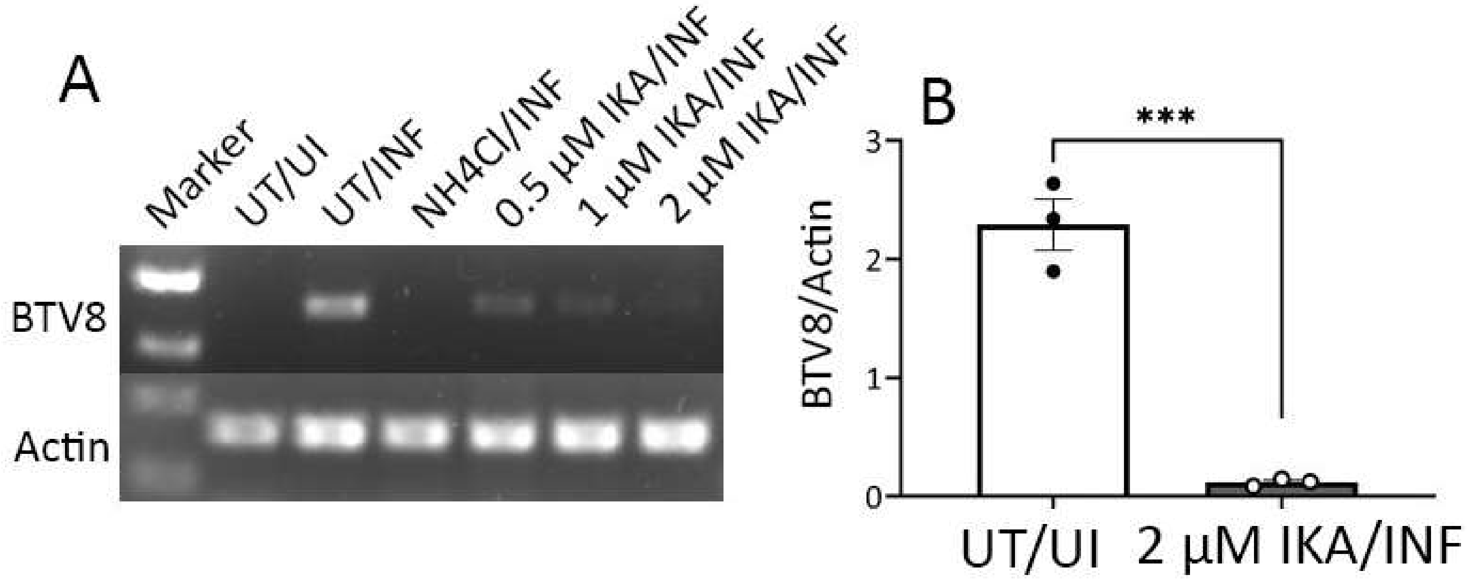

Sup. Figure 8 | Endosome alkalinization and ikarugamycin inhibit BTV-8 infection of MDBK cells. BTV cells were left untreated or pre-treated with NH4Cl (25 mM, 30 min) or ikarugamycin (0.5, 1 or 2 µM, 30 min) before infection with BTV-8 (MOI = 0.1, 1 h, in the same medium of pre-treatment). Following extensive washes with PBS supplemented with 25 mM NH4Cl, cells were incubated with growth medium supplemented with 25 mM NH4Cl for an additional 24 h. Subsequently, samples were processed for RT-PCR. (A) Representative DNA electrophoresis of RT-PCR products at 25 PCR amplification cycles. Samples are marked as: untreated-uninfected, UT/UI; untreated-infected, UT/INF; NH4Cl-treated-infected, NH4Cl/INF; ikarugamycin-treated-infected, IKA/INF (at three concentrations: 0.5 µM, 1 µM or 2 µM). (B) The graph shows the average ± SEM of the ratio of signals obtained for BTV8 divided the signals obtained for Actin (housekeeping gene) as measured by RT-qPCR.
